# Travelling to the south: Phylogeographic spatial diffusion model in *Monttea aphylla* (Plantaginaceae), an endemic plant of the Monte Desert

**DOI:** 10.1371/journal.pone.0178827

**Published:** 2017-06-05

**Authors:** Matias C. Baranzelli, Andrea Cosacov, Gabriela Ferreiro, Leigh A. Johnson, Alicia N. Sérsic

**Affiliations:** 1 Laboratorio de Ecología Evolutiva—Biología Floral, Instituto Multidisciplinario de Biología Vegetal (IMBIV), CONICET-Universidad Nacional de Córdoba, Córdoba, Argentina; 2 Department of Biology and M. L. Bean Life Science Museum, Brigham Young University, Provo, Utah, United States of America; National Cheng Kung University, TAIWAN

## Abstract

Effects of Pleistocene climatic oscillations on plant phylogeographic patterns are relatively well studied in forest, savanna and grassland biomes, but such impacts remain less explored on desert regions of the world, especially in South America. Here, we performed a phylogeographical study of *Monttea aphylla*, an endemic species of the Monte Desert, to understand the evolutionary history of vegetation communities inhabiting the South American Arid Diagonal. We obtained sequences of three chloroplast (*trnS–trnfM*, *trnH–psbA* and *trnQ–rps16*) and one nuclear (ITS) intergenic spacers from 272 individuals of 34 localities throughout the range of the species. Population genetic and Bayesian coalescent analyses were performed to infer genealogical relationships among haplotypes, population genetic structure, and demographic history of the study species. Timing of demographic events was inferred using Bayesian Skyline Plot and the spatio-temporal patterns of lineage diversification was reconstructed using Bayesian relaxed diffusion models. Palaeo-distribution models (PDM) were performed through three different timescales to validate phylogeographical patterns. Twenty-five and 22 haplotypes were identified in the cpDNA and nDNA data, respectively. that clustered into two main genealogical lineages following a latitudinal pattern, the northern and the southern Monte (south of 35° S). The northern Monte showed two lineages of high genetic structure, and more relative stable demography than the southern Monte that retrieved three groups with little phylogenetic structure and a strong signal of demographic expansion that would have started during the Last Interglacial period *(ca*. 120 Ka). The PDM and diffusion models analyses agreed in the southeast direction of the range expansion. Differential effect of climatic oscillations across the Monte phytogeographic province was observed in *Monttea aphylla* lineages. In northern Monte, greater genetic structure and more relative stable demography resulted from a more stable climate than in the southern Monte. Pleistocene glaciations drastically decreased the species area in the southern Monte, which expanded in a southeastern direction to the new available areas during the interglacial periods.

## Introduction

Quaternary climatic oscillations profoundly impacted the abundance and distribution of organisms due to changes in landscape configuration, habitats and resource availability [1]. Paleoflora reconstructions for the late Quaternary period revealed that these fluctuations, involving cold, dry glacial cycles alternating with warm, moist interglacial periods, affected biotic diversity and evolution drastically at global scale. Such impacts are relatively well studied in forest, savanna and grassland biomes, but remain less explored on desert regions of the world [2–5]. In particular, the small number of plant phylogeographic studies in arid lands, suggests that even drought-resistant plants suffered a reduction of suitable habitat due to the increased aridity and lower temperatures during Pleistocene glaciations, both in the northern (e.g. North America: [6–8]; Asia: [9, 10]) and southern hemisphere (e.g. Australia: [3]; South America: [11–15]). Moreover, some of these studies provided evidence that these climatic oscillations would have promoted allopatric divergence among isolated populations [11, 14] and, in some instances, drove speciation [9, 10].

**Fig 1:**
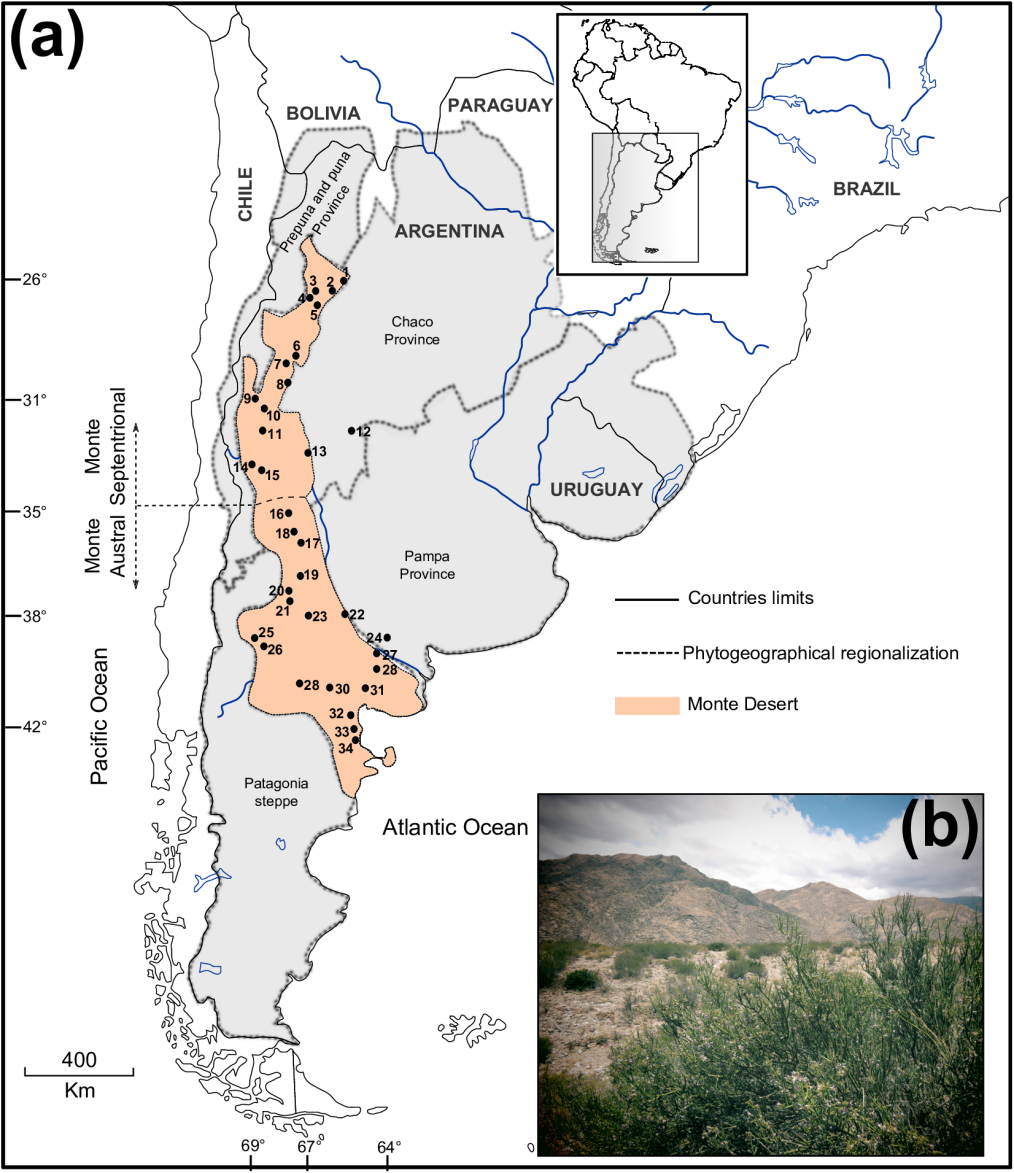
Geographical distribution of the Monte Desert and *Monttea aphylla*. **(a)** Map showing the 34 *Monttea aphylla* populations included in this study. Inset indicates the study area in South America. Locality numbers correspond to those in S1 Table. The orange area shows the extent of the Monte Desert. Thick dotted lines delimitate the phytogeographical areas. The thin dashed line represents the limit of northern and southern Monte. **(b)** Overview of the Monte Desert and a plant of *Monttea aphylla* in the foreground. The map shown in (a) is reprinted from [31] under a CC BY license, with permission from [DIVA-GIS and Dr. Robert Hijmans; see S1 Doc], original copyright [2005].

The Monte phytogeographic Province or Monte Desert, constitutes the southernmost part of the Arid Diagonal ranging from 24º 35’ S to 44º 20’ S in western Argentina [16] (Fig 1A). Interest in understand the historical processes impacting Monte Desert organisms is growing (lizards: [17–20]; mammals: [21–23]; birds: [24]). However, phylogeographic studies of plants from this area are limited to just one, based on *Munroa argentina* (Poaceae [15]; see [4]), a grass species distributed mainly in the Puna and Prepuna, overlapping though the northern Monte region. With few exceptions [19,24], all phylogeographic studies involving this region are restricted to limited areas within the Monte Desert, focusing in organisms which do not inhabit exclusively this phytogeographical area, neither considering the whole extension nor the different environmental areas within the region.

The climatic history of the Monte Desert that influenced organismal distributions in the Quaternary is complex [25]. Based on biodiversity patterns and palaeoclimatic reconstructions, Vuilleumie [26] proposed that climatic oscillations occurred in the Pleistocene were critical factors promoting differentiation and speciation of the fauna and flora in the region. Different levels of δ^18^O records in marine sediments offer a good estimation of the extent of glaciations in South America revealing several anomalies during the late Pleistocene including the Greatest Patagonian Glaciation (1.1 Million years ago, Ma), with other intense glaciations occurring 0.5 Ma, 70–60 thousands of years (Ka), and finally the Last Glacial Maximum (LGM) around 26–21 Ka. During this last glaciation period, palynological records suggest that the climate was colder, drier and windier than today in the Monte Desert [25]. For example, Markgraf [27] found around 33º to 35º S, in the current center of the Monte Desert, abundant grass pollen for the LGM, which has been interpreted as the result of the shift of the Patagonian Steppe to the north with the consequent shift of the Monte vegetation to lower latitudes [11]. Following [4,15], similar inferences could be made for the northern Monte ecotones with Puna and Prepuna, where the high altitude grasslands would have descended to lowlands during cold periods like the LGM, thus reducing the area of the Monte vegetation. In this way, the Monte vegetation would have decreased in geographic area persisting at mid-latitudes of its current extension, during glaciations, and then progressively recovered with climate amelioration following glaciations. Accordingly, one would predict that plant communities faced isolation and demographic reduction during glaciation periods, with demographic increase and range expansion during interglacial periods.

To further understand the evolutionary history of organisms of the Monte Desert and to assess the impact of Pleistocene glaciations on vegetation communities that span the southern area of the Arid Diagonal, we selected a widely distributed shrub species that occurs along the whole Monte phytogeographic Province, *Monttea aphylla* (Miers) Benth. & Hook (Plantaginaceae; Fig 1B). This species is endemic to this area extending latitudinally more than 2000 km as a dominant species within its range. The condition of endemic becomes *M*. *aphylla* an excellent proxy to infer historical process occurred in the Monte region given that its evolutionary history is inextricably linked to the history of the region. Moreover, together with other shrubs, *M*. *aphylla* is a constituent of the prevailing functional group (*i*.*e*. groups of species which share morphological and physiological attributes, use similar resources and play a similar role in the ecosystem) that characterizes this phytogeographic region [16]. Under the paleoecological context presented above, we hypothesize that *M*. *aphylla* populations were restricted to the mid-latitudes of its present distribution during the cold, dry glacial periods, with subsequent demographic increase and range expansion occurring in the interglacial cycles. To test this hypothesis, we performed explicit phylogeographical analyses and projected the species’ paleo-distribution through three different timescales using Palaeo-distribution modelling (PDM). If these glaciation cycles affected this species’ distribution, we expect to observe: 1) at mid-latitudes of the present geographical range, where the species would have persisted *in situ* during glacial cycles, higher haplotypic diversity, exclusive haplotypes and higher genetic structure than in populations located in the northern and southern edges of the species’ range; 2) an association between demographic increase and range expansion signals from mid-latitudes to the northern and southern edges during interglacial periods; 3) The PDM should reveal potential favourable and stable mid-areas of the present distribution; 4) finally, a tempo-spatial association is expected between the spatial diffusion processes and the PDM.

## Materials and methods

### Ethics statement

Plants were collected and deposited in the Herbarium of the Universidad Nacional de Córdoba, Argentina (CORD); this is a herbarium recognized and authorized by the Argentine Ministry of Environment ("Secretaría de Ambiente y Desarrollo Sustentable de la Nación"). The Argentine Administration of National Parks (APN) provided permission to collect samples in site 13 corresponding to National Park Las Quijadas (DRC237-Res.257/06 P.D. 2011, L.V. Ruiz); the Secretaría de Ambiente y Desarrollo Sustentable of Mendoza Province, provided permission to collect at sites 14–20 (Res.1087-2012, D.A. Gómez); the Ministerio de Producción of Chubut Province gave us permissions to collect sites: 33–34 (Disp.nº48/2008-2012, Sr. A.Eltceche). For the remaining collecting sites specific permissions were not required. Field studies did not involve endangered or protected species.

### Plant species and study area

*Monttea aphylla* is a leafless shrub, up to 2 meters, with green shoots. Flowers are slightly zygomorphic, violet, tubular and hermaphroditic, with a mixed reward system that offer nectar and floral-oils to pollinators; as it is strictly self-incompatible the species depends on their visits to set fruit [28]. The one seeded fruits are covered by a yellow fleshy calyx at maturity [29]. Nothing certain is known about the dispersion of the fruits, although their features suggest bird-dispersion, ants have been seen in the field carrying floral and fruit parts to their nests (Baranzelli, personal observations). The geographical range of *M*. *aphylla* closely coincides with the area proposed for the Monte phytogeographic Province; in fact, this species has allowed define the limits of the Monte Desert, specifically was used to determine the boundary between the Monte and the Espinal [16,30].

The Monte phytogeographic Province is characterized by a dry-temperate climate with an average annual precipitation of 80 to 350 mm [16,30]. Acute droughts, seasonal precipitation and topography distinguish two main ecoregions within the Monte Desert [25] (Fig 1). The northern Monte (NG), which extends from 25º-35º S, is characterized by a subtropical climate and by summer, often pelting rains; its topography is heterogeneous, dominated by mountain ridges crossed and surrounded by valleys which build a complex geography. The southern Monte (SG) stretches from 35°–44° S ruled by the Pacific winds and precipitations occurring throughout the year, while the landscape is dominated by plains, foothills and plateaus. The northernmost boundary of the Monte Desert is the Puna phytogeographical province, while their boundaries to the west is the Prepuna province, to the east are the Chaco and Pampa provinces and to the south is the Patagonian Steppe (Fig 1).

### Sampling

For genetic analyses, we sampled 272 individuals of *M*. *aphylla* from 34 populations spanning the geographical range of the species (Fig 1A). Samples were taken from eight individuals selected haphazardly at each site, separated by a minimum distance of 30 m to avoid the collection of clones or close relatives; as the species has no leaves (see Fig 1B) we collected 3 to 5 young shoots dehydrated in silica gel. For each population, geographical coordinates were obtained *in situ* with a handheld GPS. The remaining *Monttea* species, *M*. *chilensis* Gay and *M*. *schickendanztii* Griseb, *Melosperma andicola* (Benth.) Barringer and *Ourisia coccinea* (Miers) were also collected as outgroups for the phylogenetic analyses (see S2 Table).

### DNA extraction, amplification and sequencing

DNA was isolated using a modified cetyl trimethyl ammonium bromide protocol [32]. Three chloroplast intergenic spacers, *trnS–trnfM* (primers trnS^[UGA]^ and trnfM^[CAU]^ [33]), *trnH–psbA* (primers trnH^GUG^ and psbA; [33]) and *trnQ–rps16* (primers trnQ^(UUG)^ and rpL16x1; [34]), and the nuclear ITS region (primers 17SE and ITS4) were sequenced as they showed the greatest variation among several surveyed *loci*. The *trnQ–rps16* region was difficult to amplify in some samples; hence we designed primers from conserved DNA regions of samples that amplified correctly using Primer3plus (http://primer3plus.com/cgi-bin/dev/primer3plus.cgi/). We made three sets of primers, including the ends and the middle portion of the sequence (see S1 Fig and S3 Table). The protocol for chloroplast DNA (cpDNA) amplification consisted of 94°C for 3 minutes (min) followed by 30 cycles of 94°C for 1 min, 52°C for 1 min, and 72°C for 1 min, while nuclear ITS (nDNA) amplification consisted of 95°C for 2 minutes followed by 40 cycles of 95°C for 1 minute, 52°C for 1 minute, 72°C for 1 minute and 72°C for 8 min. Products were purified using USB PrepEase PCR purification plates (Affymetrix Inc, Cleveland, OH, USA), sequenced with BigDye v.3 (Applied Biosystems, Foster City, CA, USA) and purified with Sephadex (GE Health-care, Piscataway, NJ, USA) before electrophoresis on an AB3730xl automated sequencer. Electropherograms were edited using ChromasPro v.1.5 and BioEdit v7.0.9.0 [35]. Sequences were aligned with ClustalX v.1.81 [36] using default parameters and adjusted by hand. Three gaps were coded as binary characters in cpDNA sequences, and three in ITS sequences using simple indel coding [37]. All sequences were deposited in GenBank MF167912-MF167959.

### Haplotype network and molecular diversity patterns

Haplotypes were obtained using DnaSP v5 [38]; ambiguities detected at more than one site in the nDNA were resolved with the coalescent-based Bayesian method PHASE v2.1 [39] as implemented in DNAsp v5.0. Haplotype networks for each data set (i.e. nuclear and chloroplast matrix) were constructed using the median-joining algorithm implemented in Network v4.5.1.0 [40]. Ambiguous connections (*loops*) in networks were resolved using the frequency and topological criteria [41].

To statistically compare the spatial genetic patterns between genomes, we made non-parametric regression cubic splines using genetic diversity indexes (π, h and *p*) as response variables and the latitude as the independent variable. This type of regression allows a local fit of the data rather than a general adjustment as does an ordinary regression. For these analyses, we used a *gam* routine in the `*mgcv´* package [42] in R v3.0.3 [43]. Smoothing parameters were obtained by minimizing the generalized cross-validation scores [44], and Bayesian standard errors were obtained according to [42].

### Phylogenetic relationships among haplotypes and molecular dating

We reconstructed the phylogenetic relationships among haplotypes and outgroups using cpDNA data set and nDNA with phased alleles using Bayesian inference (BI) in BEAST v1.7.5 [45]. For this analysis, the models HKY+I for cpDNA matrix and GTR+I for nDNA were used according to Akaike Information Criterion (AIC), as implemented in jModelTest v2.1.4 [46]. Two independent analyzes were run for 1.5 x 10^8^ generations each one with a Yule tree prior, two Monte Carlo Markov chains (MCMC), starting with a random tree and sampling parameters every 15000 steps. Trees prior to stationary (burn-in fraction of 0.25) were excluded and convergence of estimated parameters and high effective sample sizes (ESS>200) were verified using Tracer v1.6 [47];.*log* and.*tree* files were combined using LogCombiner v1.7.5, and topologies were assessed using TreeAnnotator v1.7.5 and FigTree v1.6.1 [48].

Plantaginaceae comprises about 90 genera and 1900 species with no undisputable fossil record [49]; we therefore used published substitution rates and dates estimated from other studies (secondary calibrations) to calibrate a relax molecular clock and approximate divergence times for the main phylogroups retrieved in our study. For the cpDNA matrix, we used a uniform prior distribution of mutation rates derived from the analyses of several cpDNA spacers at the intraspecific level (0.0001–0.01 substitution per site per million years; based on [50]). For the nDNA, we implemented a lognormal prior distribution based on a published substitution rate for *Plantago* (0.000427 ± 0.00098 substitution per site per million years; [51]), a genus belonging to the same family as *Monttea*. This rate value is intermediate between the lowest (0.00038 subs/site/Ma, reported for the shrub *Hamamelis*) and the highest (0.0083 subs/site/Ma, reported for the herb *Soldanella*) ITS substitution rate surveyed across Angiosperms [52]. In addition, the split of the stem lineage of the Tribe Melospermae (*Monttea* + *Melosperma*) from *Ourisia*, *i*.*e*. the root of the tree, was set to 30 Ma (Standard Deviation = 10), following the estimation of [49].

### Population genetic structure

We estimated the optimal number of populations with the Bayesian program Geneland v4.0.3 [53]. We ran five independent replicate runs with the number of populations ranging between 1 and 12, and assuming a correlated allele frequencies model and a spatial model without uncertainty on coordinates. Each run consisted of 1 x 10^7^ iterations, a thinning interval of 1000 and a burn-in phase of 200 iterations. To assess convergence among runs, burn-in plots of the posterior probability parameters were visualized and compared the posterior estimates of the number of populations. Probability maps of population membership on a spatial domain defined by 100 pixels in both X- and Y-axes were performed.

For each locality and for each Geneland genetic groups the following diversity indices were calculated independently on cpDNA and nDNA data sets: number of polymorphic sites (S), number of haplotypes (k), nucleotide diversity (π), haplotype diversity (h) and mean number of pairwise differences (p) using DnaSP v5. Standard errors were estimated using bootstrap resampling (10,000 pseudoreplicates).

### Demographic history analyses

For each detected genetic group we used several approaches to infer past demographic process for each dataset (i.e. nDNA and cpDNA). First, we calculated Tajima’s D and Fu’s FS [54,55] neutrality tests. These tests assume that populations have been for long periods in mutation–drift balance. When this is not true due to sudden expansion, these indices usually have negative values. Significance for both values was determined from 10000 simulated samples under a standard coalescent neutral model.

Second, we obtained the mismatch distribution of pairwise differences among individuals, comparing this with a model of population expansion. Goodness of fit of the observed mismatch distribution to that expected under a sudden expansion model was evaluated with the sum of squared deviations (SSD) using parametric bootstrapping (10,000 replicates). These analyses were performed in DnaSP v5 and Arlequin v3.11 [56].

To complement mismatch distributions and estimate timing of demographic events, Bayesian Skyline Plot (BSP) was run in BEAST v1.7.5. This analysis allows estimation of population size changes over time without *a priori* specification of a particular model. Since BSP was designed for a single locus, independent analyzes were run for each dataset (i.e. nDNA and cpDNA) and for each genetic group recovered. The number of group intervals was set to 10, the Bayesian skyline analysis was performed in the piece-wise constant model and the maximum time in the root height was set on Median. For these analyses, the HKY model was used for the cpDNA N-1 and N-2 and for the nDNA N-2; HKY+I for nDNA N-1, HKY+I+G was used for both data sets for the southern Group (see Results) according to AIC criteria implemented in JmodelTest. Two MCMC starting with a random tree were run for 1 x 10^8^ generations, with parameters sampled every 10000 steps. The same parameter settings for clock rate, clock model, chain convergence check and tree annotation were used as previously described for the BI haplotype tree reconstruction. BSPs were visualized using Tracer v1.6.

### Bayesian spatio-temporal diffusion analyses

As an integrative approach, for those genetic groups that evidenced clear signals of population growth via the previous demographic analyses, we reconstructed the geographical origin and the spatial distribution of lineages during diversification using a continuous diffusion model through space (“Relaxed Random Walk”, RRW; [57]) implemented in BEAST v1.7.5. These diffusion models, using the geographic coordinates of each tip in the genealogy, infer the distribution of ancestors and the diffusion of lineages continuously over space and time while considering for genealogical uncertainty [57]. Likewise, these Bayesian methods of phylogenetic inference allow estimating and comparing demographic and geographic expansion models with enough power to distinguish between them [39]. In the case of continuous-diffusion models, the spatial expansion rate is variable along the tree branches, based on analogous models to those implemented for relaxed-molecular clocks [58] and which is appropriate for species with large distribution ranges where it is expected that the spread through space has not occurred evenly [57]. This flexible phylogeographic approach which simultaneously accomplishes both pattern inferences and model testing, resulted in novel insights about the spread of several viral epidemics (e.g. [59]), and more recently has been applied to the study of diffusion dynamics of slower evolving organisms [60, 61]. To our knowledge this approach has not been previously used in plants at a geographic scale.

Independent analyzes were run for each dataset (i.e. nDNA and cpDNA). In each analysis, we used the same setting parameters for nucleotide substitution models, clock rate, clock model, chain convergence check and tree annotation described for the previous bayesian analyses, but for this analysis a population coalescent Bayesian Skyride model for the prior tree, and a normal distribution for the diffusion rate were set. We manually modified.*xml* files to include geographic uncertainties in the sequences having the same geographical coordinates (i.e. belong to the same location) using the option *jitter* on statistical *TraitLikelihood* with a parameter of 0.01. To summarize the posterior distribution of ancestral locations using the RRW model, we annotated nodes in a maximum clade credibility tree (MCC) using the program TreeAnnotator v1.7.5. This MCC obtained under the continuous diffusion model was then used as an input in SPREAD3 v0.9.7-1rc [62] to analyze and visualize lineage diversification through the landscape. To obtain convergence (ESS>200), we subsampled each data set selecting only one individual representing each haplotype found in the corresponding localities (e.g. [20,60]).

### Palaeo-distribution modelling

Using Herbarium records (CONC, CORD, BAB, MERL, SI, CTES, DAO, IBONE, LIL), global databases (ASU, GBIF) and records from field trips between 2009–2014, we obtained 93 trustable presence points for *M*. *aphylla*. Moreover, during field trips we selected 95 “absence records”, sites along the species distribution and their boundaries where the species was systematically absent during field trips.

Climatic data with a resolution of 2.5 arc minutes (5 km^2^) for current and past conditions were downloaded from the WorldClim database v1.4 [31] and represented by 19 bioclimatic variables derived from the monthly temperature and rainfall values. After downloading, all bioclimatic layers were cropped to span from 26.07° S to 42.60° S and from 69.81° W to 63.52° W, a spatial range that corresponds to the current Monte Desert. We performed the distribution model for current conditions, and then projected onto palaeoclimatic models for the Last Interglacial period (LIG; 130–114 ka; [63]) and two models for the LGM (21 Ka), the Community Climate System Model (CCSM) and the Model for Interdisciplinary Research On Climate (MIROC). Since these models present different initial configurations, they simulate different climatic conditions for the LGM [64]. For these analyses, we used the Maximum Entropy algorithm in MaxEnt v3.3.3 [65] and DIVA-GIS v7.5 [63]. We selected the autofeatures option in MaxEnt, which allows for linear, quadratic, product, threshold, and hinge features to describe relationships between specimen locations and environmental conditions.

Before projecting the species distribution into paleoclimatic scenarios we conducted a phase tuning where several tests were made to avoid overestimating the potential distribution of *M*. *aphylla* under current environmental data. It is important to get optimal models to achieve a balance between complexity (i.e. number of parameter used) and realism (i.e. predictive ability of the distribution of the species under study), and this is even more relevant when projecting onto another set of climate data, such as layers of the LIG and LGM (i.e. transferability; [66]). Therefore, first, Pearson correlations were performed between climatic variables to reduce the effect of collinearity by eliminating those highly correlated (r> 0.85). Second, following the recommendations of [66] we tested 12 values for the regularization multiplier (β, from 0.25 to 3.0, in increments of 0.25) which represented a penalty for each term included in the model [66]. For each value of β, we ran MaxEnt using the following settings: random test percentage = 25; convergence threshold = 0.00001; maximum iterations = 1000 and 10 replicate run type = crossvalidate. We used different statistical parameters in MaxEnt output (i.e. the lowest values of average differences between training and test data set, the average Area Under the Curve of the Receiver Operator Characteristic Function (AUC) and the shape of response curves) to evaluate model performance for each value of β. As reported in other studies (e.g. [20]), the species-specific smoothing increased model quality and provided more confidence for our projections to the past climate scenarios. After selecting the bioclimatic variables to include in the model, and the best value of β, final models were run and projected on past climate scenarios using the same parameters as in the phase tuning.

We evaluated model performance with statistics commonly used for judging the performance of species distribution models: the AUC, the true skill statistic (TSS), the proportion of presences and absences correctly categorized (PCC), the proportion of presences correctly predicted (sensitivity), and the proportion of absences correctly predicted (specificity). The first statistic was calculated in MaxEnt, based on the presence points and pseudo-absence generated by this algorithm [65]. For the other four statistics, we used the converted the continuous values of the current distribution model to a binary variable that represents the presence or absence of suitable climate, and then we evaluated how well presence and absence were predicted by the model [67]. To determine the threshold value for each prediction, we used the lowest value of probability of occurrence obtained among the 93 trustable points of presence used in the model. Finally, a jackknife test was conducted to assess the relative importance of each environmental variable included.

## Results

### DNA sequence matrices

The chloroplast DNA intergenic spacers *trnS–trnfM*, *trnQ–rpL16* and *trnH–psbA* included 725, 1025 and 551 characters, respectively (2301 in total), providing a total of 23 variable sites (1%); the total number of sequenced specimens for the cpDNA was 272. Because the chloroplast is typically inherited uni-parentally without recombination, these three regions were *a priori* combined into a single matrix. The nuclear ITS region included 681 characters of which 18 were variable sites (2.64%); the total number of sequenced individuals was 269, however, because of the presence of heterozygotes, the total number of alleles was 301.

### Haplotype network and molecular diversity patterns

**Fig 2:**
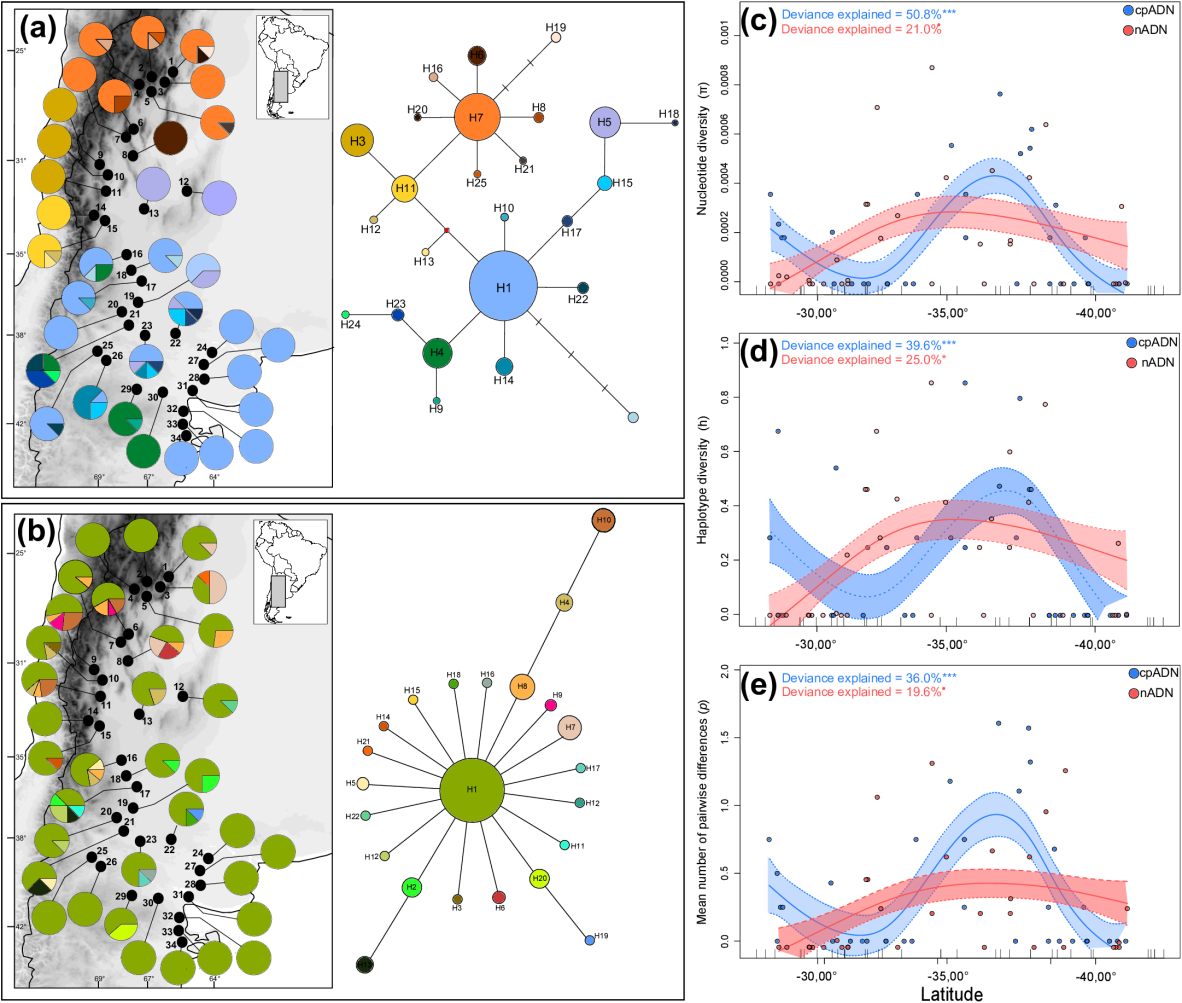
**Geographical distribution, genealogical relationships and spatial patterns of genetic diversity for (a) the chloroplast and (b) the nuclear haplotypes, recovered from 34 populations of *Monttea aphylla*.** On the maps, pie charts reflect the frequency of occurrence of each haplotype in each population. Haplotype colours correspond to those shown in the networks to the right. In the networks, haplotype are designated by numbers, and circle sizes are proportional to haplotype/allele frequencies. Diamond represents a missing intermediate haplotype not observed in the analyzed individuals. **(c-e)** Non-parametric regression cubic spline using genetic diversity indexes (π, h and p) as response variables and the latitude as the independent variable, performed for chloroplast and nuclear data sets. Dotted lines show ±1 Bayesian standard error intervals (N = 34 locations). The maps shown in (a, b) are reprinted from [31] under a CC BY license, with permission from [DIVA-GIS and Dr. Robert Hijmans; see S1 Doc], original copyright [2005].

From the cpDNA sequenced specimens, 25 haplotypes were obtained. Genealogical relationships among haplotypes and their geographical distribution are shown in Fig 2A. The haplotype network showed that three haplotypes, H1, H7 and H3 were the most frequent found in 47%, 20.5% and in 8.8% of the sampled sites, respectively; each was restricted to a particular geographic region following a latitudinal pattern: H1 prevailed in most localities south of 34° S, distributed over the entire southern Monte—this haplotype forms the core of the "star-like" topology of the southern haplotypes; the second most frequent haplotype, H7, was restricted to the northern range of the species at latitudes lower than 30° S, also forming an internal node from which haplotypes all exclusively distributed in the northern Monte are derived; finally, H3 is an external haplotype derived from H11 both restricted to an area between 30° S and 33° S, in the southwest of the northern Monte (Fig 2A). Sites with higher haplotype diversity were found mainly in two areas, one located at the northern edge of the northern Monte and the other one at the northern margin of the southern Monte (mid-latitudes), while 18 monomorphic sites were mainly distributed to the southern edge (Fig 2A).

From the nDNA region 22 haplotypes were obtained; the haplotype network was less structured than the previous one and haplotypes were arranged in a “star-like” topology; H1 was the most frequent and the only haplotype found in all sampled sites (Fig 2B); the following more frequent haplotype was H8 found in 7 populations (20.58% of the sampled sites) all located at latitudes lower than 35º S; 10 haplotypes were exclusive, mostly derived from H1. Sites with higher haplotype diversity were found mainly in the mid-northern area of the species distribution, while the 13 monomorphic sites containing H1 were marginally distributed, especially at latitudes higher than 39°S (Fig 2B).

Overall molecular diversity indices for both markers showed the highest haplotype diversity at mid latitudes (between 35º and 38º S), partially decreasing to the extremes of the species distribution. This pattern is concordant with results obtained from the regressions performed between genetic diversity indices and latitude for each used molecular marker, which show zones of higher genetic diversity between 35° S and 40° S for the nDNA, followed by two zones of lower genetic diversity between 30° S and 35° S, and at latitudes higher than 40°S for the cpDNA (Figs 2C–2E).

### Phylogenetic relationships among haplotypes and molecular dating

**Fig 3:**
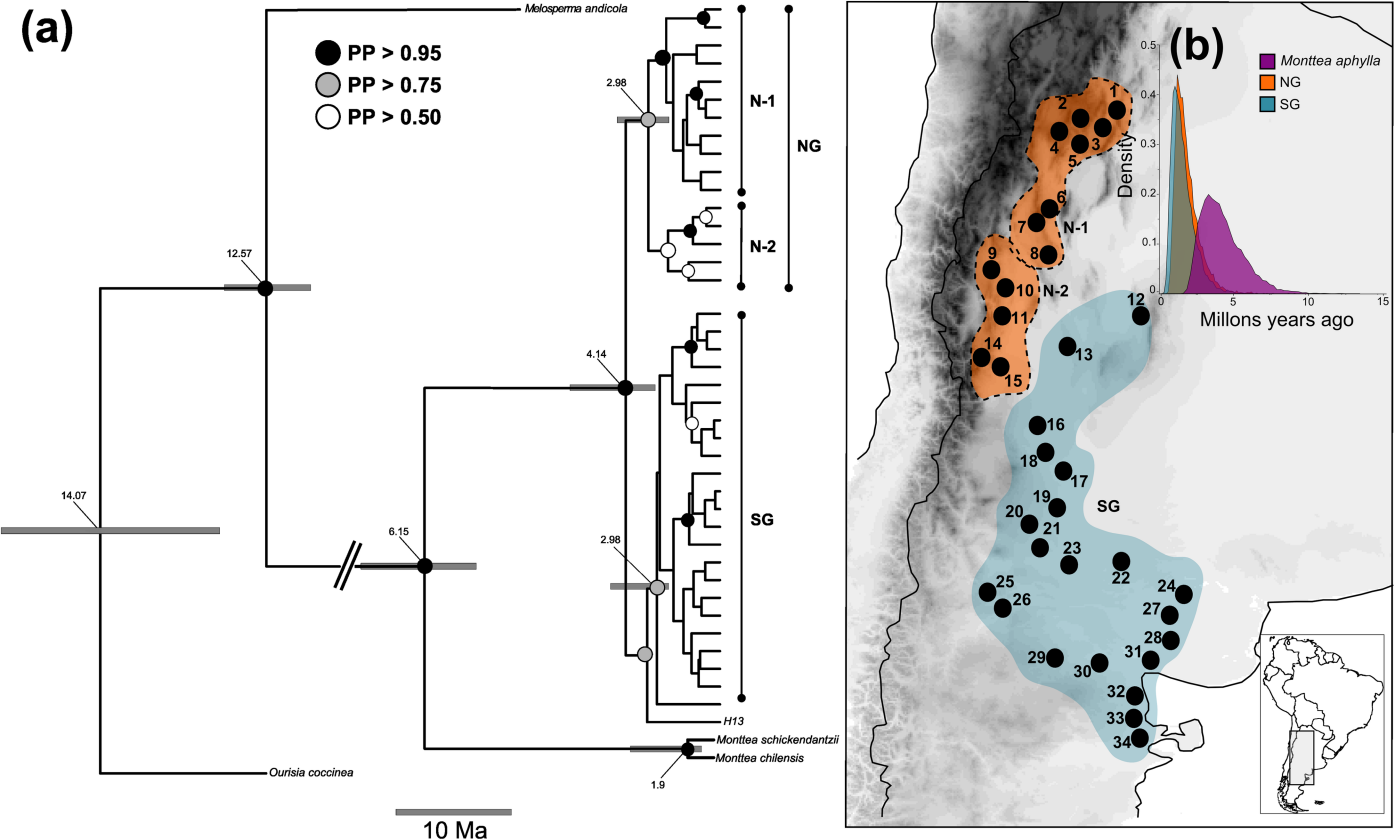
**Evolutionary relationships of *Monttea aphylla* lineages (a)** Maximum clade credibility tree of *Monttea aphylla* from cpDNA (*trnS–trnfM*, *trnQ–rpL16* and *trnH–psbA* regions) and nDNA (ITS region) reconstructed using bayesian inference. Posterior probability (PP) values are indicated by node colors and nodes with no indicative of support have pp < 0.50. Numbers given for each node are the estimated (median) divergence time in million years, and grey bars indicate the 95% highest posterior densities over the median value. **(b)** Posterior density plots of divergence times for *Monttea aphylla* and each detected genetic group. NG: northern Monte. N-1, N-2: main detected lineages in northern Monte. SG: southern Monte. The map shown in panel (b) is reprinted from [31] under a CC BY license, with permission from [DIVA-GIS and Dr. Robert Hijmans; see S1 Doc], original copyright [2005].

The Bayesian phylogenetic reconstruction performed with both data set partitions retrieved all *M*. *aphylla* haplotypes nested in a strongly supported clade which include two subclades with moderate supports corresponding to the northern and southern Monte (0.80 and 0.76 posterior probability, respectively; Fig 3). The initial diversification of *M*. *aphylla* was estimated at 4.14 Ma [95% highest posterior density (HPD) = 1.07–8.34] while diversification of haplotypes within the northern Monte began at 2.97 Ma (95% HPD = 1.65–6.27 Ma). The individual with H13 haplotype of cpNDA, located at the southwestern corner of northern Monte, appears as the sister lineage of a clade distributed exclusively in the southern Monte (Fig 3). This southern clade, comprising all haplotypes derived from the most frequent H1 in cpDNA, was retrieved as a group with little phylogenetic structure, corresponding to the "star-like" topology observed in the chloroplast network. Diversification within this southern clade would have started around 2.98 (95% HPD = 0.7–6.05 Ma; Fig 3).

### Population genetic structure

**Fig 4:**
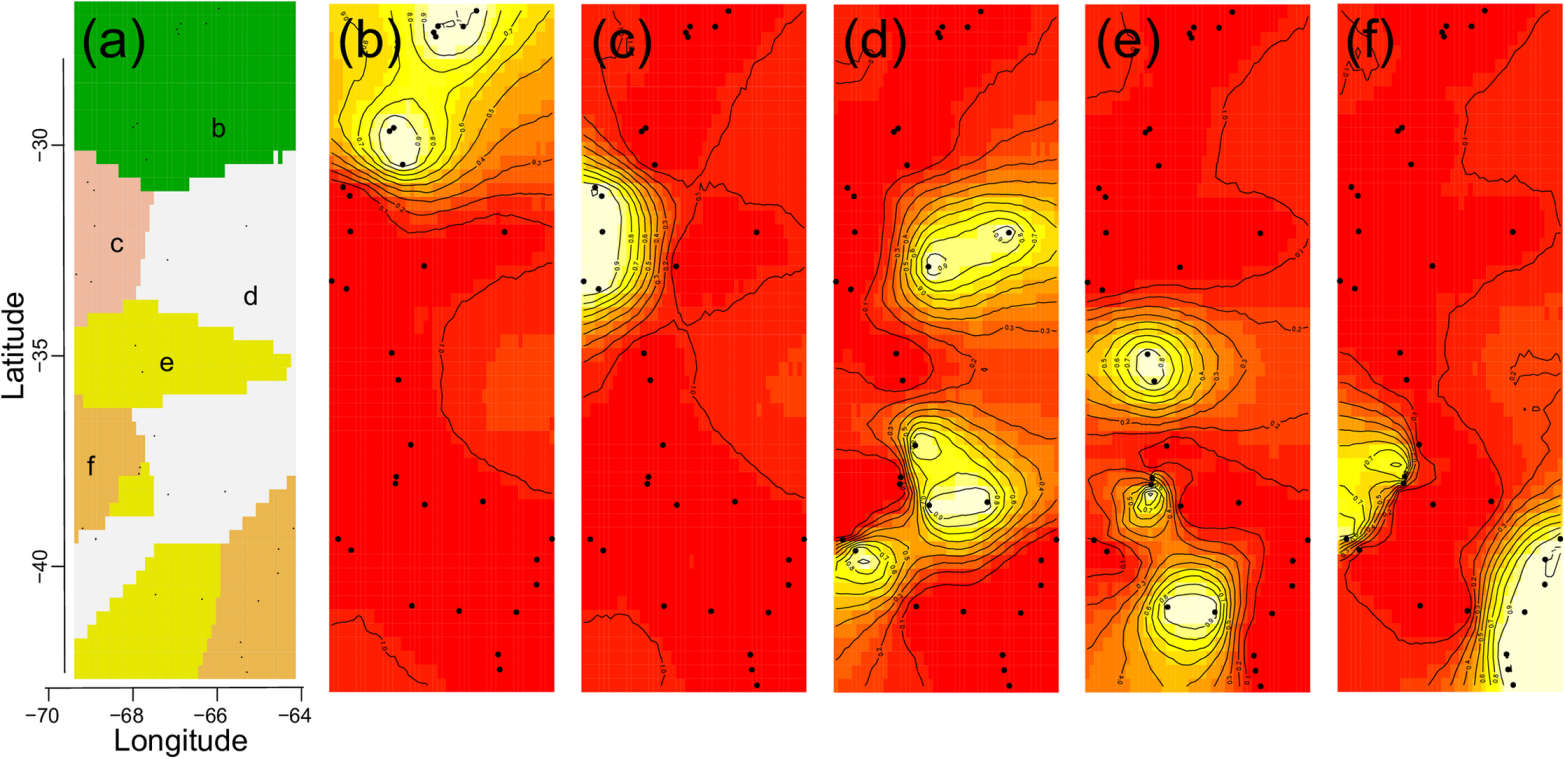
Maps of posterior probabilities of cluster membership based on Geneland analyses. Panel **(a)** shows a synthetic map of the mode of the posterior probability distribution for each pixel belonging to each inferred population. Letters (b-f) indicate population labels. Panels (**b**–**f)** show maps of the study area with the relative posterior probability of belonging to each of the five inferred populations: black dots represent the geographical position of sampled locations, and lighter colour reflects a higher posterior probability of membership to different clusters.

The Bayesian population structure analyses performed with Geneland retrieved five clusters following a predominant latitudinal pattern (Fig 4A). Posterior probability maps for the distribution of these five clusters are shown in Fig 4B–4F; two groups were located in northern Monte (N-1 and N-2) and three in the southern Monte (S-1, S-2 and S-3). N-1 comprised eight localities in the northern part of the geographical range (Fig 2B; 26°– 30° S, 65°– 67° W), while N-2 included five localities at the limit with the Precordillera, located along valleys in a small longitudinal fringe (Fig 4C; 30°–33° S, 67°–68° W). In the southern Monte, S-1 comprised two sites at the easternmost locations of the species in the Monte-Chaco ecotone and four localities in the southwest of the species distribution (Fig 4D; 31°–39° S, 65°–68° W); the S-2 group consisted of six localities in the south-central area of the species’ distribution, at the border between the Monte and the Patagonian steppe (Fig 4E; 33°–40° S, 65°–68° W); finally, S-3 included ten localities following a diagonal from northwest of the southern Monte to southeast, near Valdez peninsula in the Atlantic Ocean (Fig 4F; 37°–42° S, 65°–69° W).

### Demographic history analyses

For genetic groups located at both extremes of the geographical distribution of the focal species (i.e. the SG, S2 and S3 and N1) all demographic analyses showed evidence of recent demographic expansion based on chloroplast and nuclear markers, as indicated by the negative and significant values for Fu‘s and Tajima’s neutrality tests, and by the SSD index, which indicates that the observed mismatch distribution did not differ from an expected sudden expansion model (Tables 1 & 2). The S1 group also showed consistent evidence of recent demographic expansion based on neutrality tests and mismatch distribution analyses but only based on nDNA data set (Table 2).

**Table 1:** Diversity indices and results of demographic analyses in Monttea aphylla cpDNA lineages performed derived from spatial analysis of Geneland. polymorphic sites (S), number of haplotypes (K), nucleotide diversity (π), haplotype diversity (h), mean number of pairwise differences (p), Tajima’s D, Fu’s FS and sum of squared deviations (SSD).

Ecoregión	Genetic Groups	Diversity indices						Demographic analyses		
		Nsec	S	K	π (± SD)	h (± SD)	*p* (± SD)	D	FS	SSD
Monte Septentrional	N-1	64	7	7	0.0002 (± 0.0002)	0.402 (± 0.072)	0.4702 (± 0.4153)	-1.72366*	-4.599**	**0.0017**
	N-2	40	7	8	0.0003 (± 0.0002)	0.529 (± 0.052)	0.6423 (± 0.5105)	-0.07567	-0.4107	0.0158.
Monte Austral	S-1	48	2	4	0.0005 (± 0.0003)	0.623 (± 0.052)	0.9252 (± 0.6546)	1.9378	0.660	**0.0133**
	S-2	40	7	8	0.0005 (± 0.0003)	0.691 (± 0.055)	1.0512 (± 0.7128)	-1.0001*	-3.178*	**0.0094**
	S-3	80	4	3	0.00005 (± 0.00009)	0.050 (± 0.034)	0.1000 (± 0.1723)	-1.8083**	-2.420**	**0.0008**
	SG	168	12	13	0.0005 (± 0.0003)	0.593 (± 0.039)	1.0605 (± 0.7060)	**-1.2303**.	-4.857**	**0.0058**

Results consistent with range expansion are indicated in bold. Genetic groups where range expansion is observed consistently in all analyzes are shown in grey.

P<0.1

*P<0.05

** P<0.001.

**Table 2:** Diversity indices and results of demographic analyses in Monttea aphylla nDNA lineages performed derived from spatial analysis of Geneland polymorphic sites (S), number of haplotypes (K), nucleotide diversity (π), haplotype diversity (h), mean number of pairwise differences (p), Tajima’s D, Fu’s FS and sum of squared deviations (SSD).

Ecoregión	Genetic Groups	Diversity indices						Demographic analyses		
		Nsec	S	K	π (± SD)	h (± SD)	*p* (± SD)	D	FS	SSD
Monte Septentrional	N-1	64	6	7	0.0008 (± 0.0007)	0.406 (± 0.073)	0.5842(± 0.4762)	**-1.2807.**	-3.6072*	**0.0024**
	N-2	46	5	6	0.0009 (± 0.0008)	0.317 (± 0.088)	0.6415 (± 0.5087)	-1.0757	-2.5171	**0.0180**
Monte Austral	S-1	50	8	8	0.0005 (± 0.0006)	0.329 (± 0.086)	0.4351 (± 0.3977)	-2.0639**	-7.0254**	**0.0004**
	S-2	41	6	7	0.0011 (± 0.0009)	0.490 (± 0.091)	0.7878 (± 0.5840)	**-1.1630.**	-3.1282*	**0.0114**
	S-3	93	5	7	0.0008 (± 0.0008)	0.361 (± 0.063)	0.5698 (± 0.4672)	-0.9014	-3.3349*	**0.0025**
	SG	187	13	17	0.0008 (± 0.0008)	0.380 (± 0.046)	0.5942 (± 0.4777)	-1.8317**	-18.0353**	**0.0005**

Results consistent with range expansion are indicated in bold. Genetic groups where range expansion is observed consistently in all analyzes are shown in grey.

P<0.1

*P<0.05

** P<0.001.

**Fig 5:**
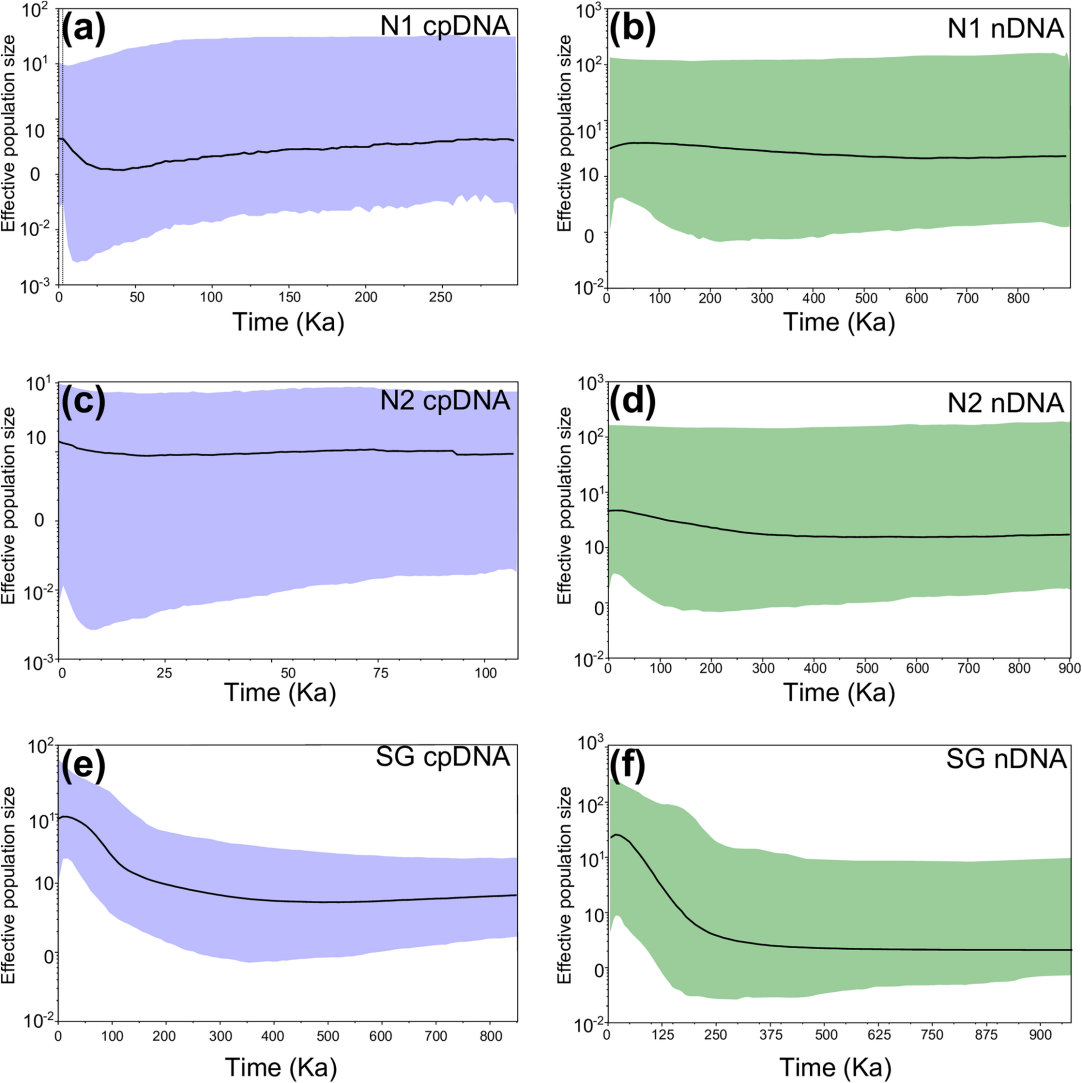
Variation through time in effective population size in *Monttea aphylla*. Results based on Bayesian Skyline Plot for each detected genetic groups and for each data set, cpDNA (left column) and nDNA(right column). **(a-b)** For the N-1 group **(c-d)** for the N-2 group **(e-f)** for all localities of southern Monte. The y-axis represents effective population size expressed on a logarithmic scale. Bold lines give the median of effective population size through time and the gray lines represent the 95% highest posterior densities over the median estimates along the coalescent history of the lineages.

The BSP was run independently for the two genetic groups of the northern Monte (N-1 and N-2), while BSP was evaluated jointly for all localities of the southern Monte because the group was retrieved as monophyletic and and the three genetic groups retrieved by Geneland shared haplotypes among them. Results showed congruent patterns between nuclear and chloroplast signals; outcomes evidenced a constant population size over the last 250–800 Ka for N-1 group, and 100–900 Ka for N-2 group (Fig 5A–5D). The BSP for southern Monte sampling sites showed an increase in the effective size that would have begun about 100–250 Ka, and lasted until the beginning of LGM, when a return to relative stability of the population size was observed (Fig 5E and 5F). Thus, the final mean population size turned to be 10 times higher than before the expansion (Fig 5E and 5F).

### Bayesian spatio-temporal diffusion analyses

Considering the results of demographic analyses, the four approaches used retrieved clear signals of recent demographic expansion in the southern edge of *M*. *aphylla* distribution, while for the N-1 genetic group in the northern Monte, neutrality tests and mismatch distribution evidenced signals of relatively recent demographic expansion. Therefore, the phylogeographic history of spatial diffusion was inferred for the lineage located in the southern Monte and N-1 in the northern Monte.

**Fig 6:**
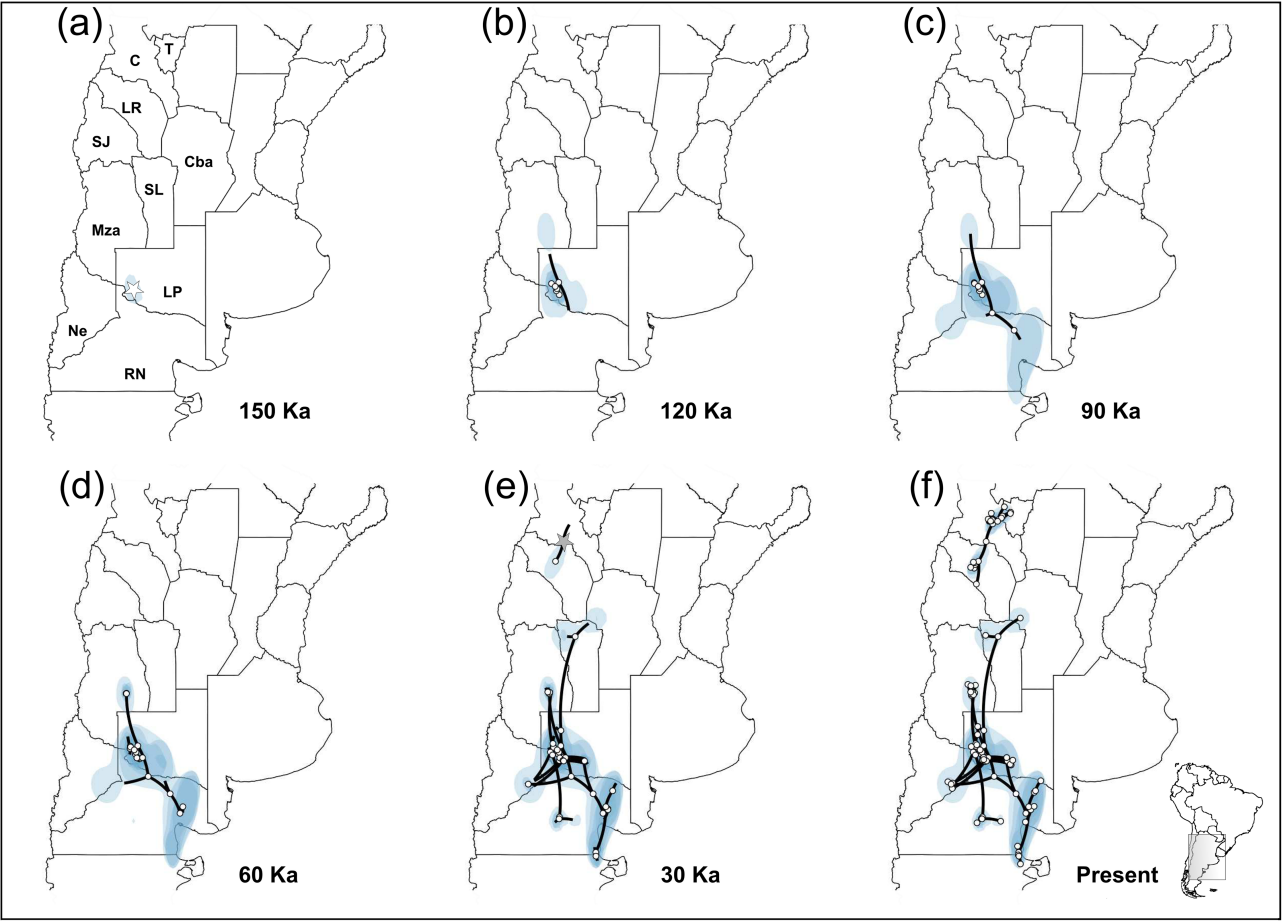
Bayesian spatiotemporal diffusion analysis of *Monttea aphylla* cpDNA lineages. Spatial projection of the diffusion pattern through time, based on the maximum clade credibility tree (MCC), estimated with a Bayesian phylogeographic analysis in BEAST (RRW model) at six time slices: **(a)** 150 Ka. **(b)** 120 Ka. **(c)** 90 Ka. **(d)** 60 Ka. **(e)** 30 Ka. **(f)** Present time. The lines represent the branches of the MCC tree. Blue areas represent the 80%-HPD uncertainty in the location of ancestral branches with a gradient from light to dark representing older vs. younger diffusion events. Locations of the expansion origin of each lineage are indicated with starts (gray for N-1 and white for SG). The maps shown are reprinted from [31] under a CC BY license, with permission from [DIVA-GIS and Dr. Robert Hijmans; see S1 Doc], original copyright [2005].

For cpDNA matrix, the spatial diffusion rate for the southern Monte group was 698.000 km/Ma (95% HPD = 354.184–1281.901). The RRW diffusion model inferred that the expansion of the southern group would have begun at around 150–180 Ka and inferred its, geographic origin in southwestern La Pampa Province bordering with Río Negro river, in the north of southern Monte (37°12'–38°18' S, 67°48'– 66°38' W; Fig 6A). The spatiotemporal reconstruction of chloroplast lineage diversifications, indicates that the southern lineage of *M*. *aphylla* experienced four main colonization phases: (1) around 120 Ka a short distance colonization to the southeast established the ancestors of the current locations of La Pampa Province (Fig 6B); (2) from there, at around 90 Ka, the cpDNA patterns revealed a second colonization to the southeast into southeastern Río Negro Province (Fig 6C); (3) cpDNA lineages, increasingly advanced towards the southeast, reaching the northern areas of Valdez Peninsula at around 60 Ka (Fig 6D); (4) before and during the LGM (ca. 21 Ka), expansions continued (Fig 6E), being more intense during the late Pleistocene and Holocene enlarging previously colonized areas. The highest diversification process was recorded during the LGM, including colonizations to the north of San Luis and southwest of Córdoba Province, giving rise to the current geographic distribution range for southern Monte groups in *M*. *aphylla* (Fig 6F).

For the N-1, the spatial diffusion rate was 457.776 km/Ma (95% HPD = 330.002–599.370) based on the cpDNA matrix. The origin of the expansion revealed by the cpDNA would have began 50 Ka, around the northwestern area of La Rioja Province (26°16'– 29°29' S, 67°34'– 66°16' W; Fig 6E). Then, since 30 Ka to the present, the cpDNA revealed short distance colonizations to the northeast and southwestern areas of the northern Monte (Fig 6E).

**Fig 7:**
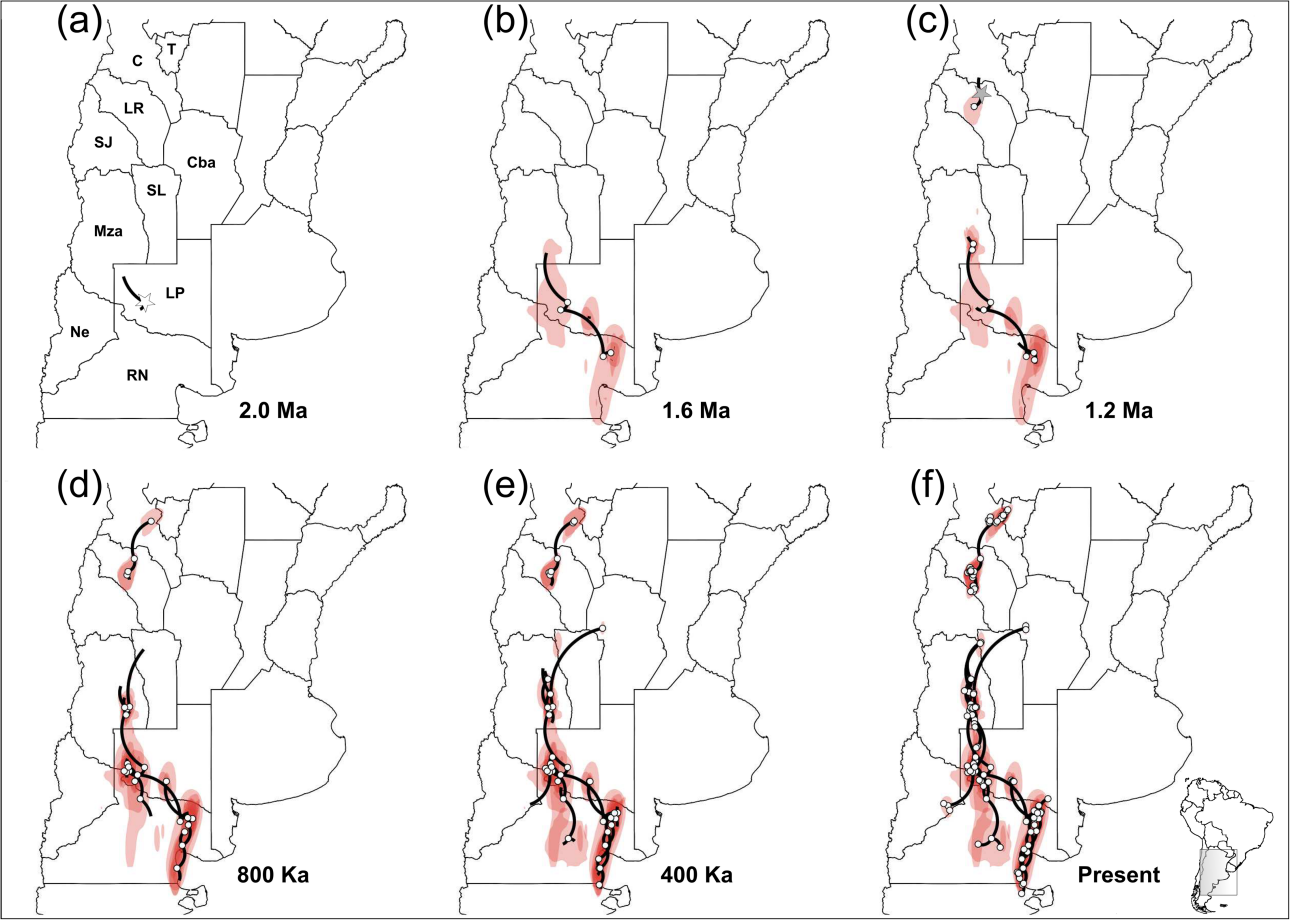
Bayesian spatiotemporal diffusion analysis of *Monttea aphylla* nDNA lineages. Spatial projection of the diffusion pattern through time, based on the maximum clade credibility tree (MCC), estimated with a Bayesian phylogeographic analysis in BEAST (RRW model) at six time slices: **(a)** 2 Ma. **(b)** 1.6 Ma. **(c)** 1.2 Ma. **(d)** 800 Ka. **(e)** 400 Ka. **(f)** Present time. The lines represent the branches of the MCC tree. Red areas represent the 80%-HPD uncertainty in the location of ancestral branches with a gradient from dark to light representing older vs. younger diffusion events. Locations of the expansion origin of each lineage are indicated with starts (gray for N-1 and white for SG). The maps shown are reprinted from [63] under a CC BY license, with permission from [DIVA-GIS and Dr. Robert Hijmans; see S1 Doc], original copyright [2005].

For nDNA matrix, the spatial diffusion rate for the southern phylogroup was 179.862 km/Ma (95% HPD = 0.03–494.844). The origin of the expansion showed great uncertainty and would have begun 2 Ma between western La Pampa Province and eastern of Río Negro Province, (35° 03'– 42°20' S, 68°39–63°49' W; Fig 7A). From these places, the spatiotemporal reconstruction of the southern lineages diversification showed subsequent colonizations to the northern and southern areas of the southern Monte: (1) to the southeast of Mendoza Province at around 1.6 Ma (Fig 7B), (2) to the north of San Luis Province around 1.2 Ma (Fig 7C), (3) the western Cordoba Province 800 Ka (Fig 7D). In the same period, and till 400 Ka,a expansions would have continued to the south near to Peninsula de Valdez (Fig 7E and 7F).

For the N-1, the spatial diffusion rate was 121.759 km/Ma (95% HPD = 0.007–334.292) and the origin of the diffusion revealed by the nDNA began around 1.6 Ma in the center of La Rioja Province (28°23'– 30° 09' S, 68° 05'– 67°11' W; Fig 7B). Then, since 1.2 Ma to the present, the nDNA revealed short distance colonizations to the northeast of the northern Monte (Fig 5C–5F).

### Palaeo-distribution modelling

Based on Pearson correlations, 10 out of the 19 bioclimatic variables were selected and the best model optimization for the current climatic conditions was obtained with β = 1.5 and the AUC average value obtained for the model was 0.810 (± 0.061; see S4 Table). To the same model, it was observed that the sensitivity of models was 0.946 ± 0.041, and values of specificity were also high (0.789 ± 0.036). In the same way, the PCC was 0.867 ± 0.055 and the TSS was 0.816 ± 0.062 (see the curated database of the distribution–presence and absence–of *M*. *aphylla* S5 Table). All these statistics indicated good performance of the MaxEnt algorithm, allowing a reasonable projection onto a paleoclimate data.

**Fig 8:**
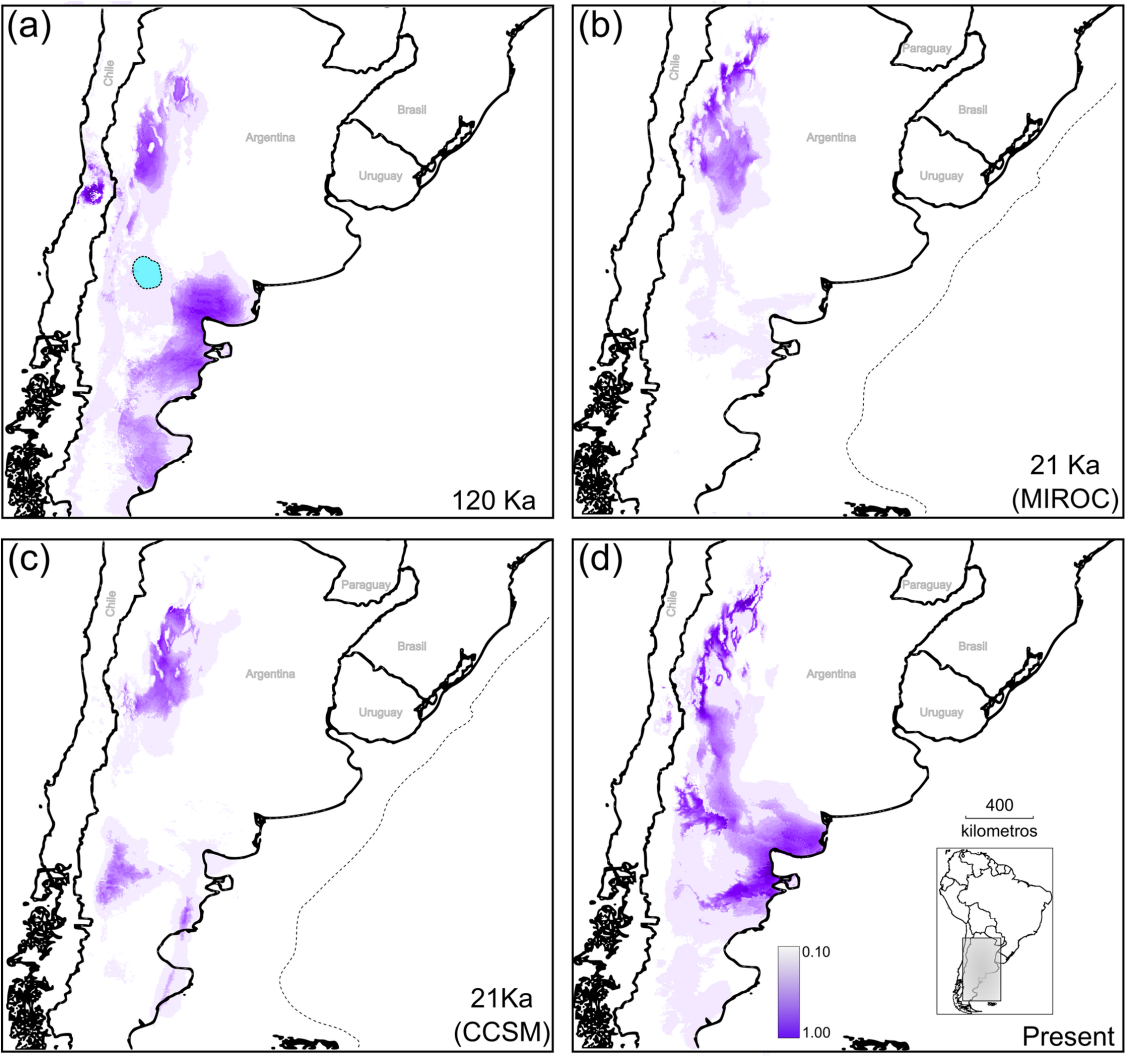
Changes in the potential distribution of *Monttea aphylla* across Quaternary climatic fluctuations and present climatic. Potential distributions are shown in gray, in all models darker shading indicates most favourable areas as averaged across 10 cross-validation runs; green shades represent topography, being darker shades higher elevations **(a)** Potential distribution during the Last Interglacial maximum (LIG). Light-blue polygon over the LIG prediction represent 80% HPD uncertainty for the location of the expansion origin for cpDNA (see Fig 6). Potential distribution during the Last Glacial Maximum (LGM) under CCMS **(b)** and under MIROC **(c)** models. **(d)** Current potential distribution. The dotted lines in b and c represent the extent of land area above sea level, which included the Atlantic continental shelf at the LGM. The maps shown are reprinted from [31] under a CC BY license, with permission from [DIVA-GIS and Dr. Robert Hijmans; see S1 Doc], original copyright [2005].

The paleo-distribution obtained for *M*. *aphylla* during the LIG (120 Ka) suggests a slight disjunction in relation to its current geographical range, with areas of high probability of occurrence in the northern and southern edges of the distribution connected by areas of moderate to low probability along mid-latitudinal areas of the Monte (Fig 8A). Both LGM models (CCSM and MIROC) showed a reduction of high probability areas being mainly restricted to the northern Monte (Fig 8B and 8C); the probability of occurrence projected by MIROC for the southern Monte is equal to or less than 10%, while CCSM, also predicted areas but with moderate probability of occurrence for the southwestern part of the range, i.e. in the current ecotone Monte-Patagonia (Fig 8B). Neither models support the presence of *M*. *aphylla* over the current Atlantic coast and mid-latitudes of the southern Monte during the LGM with high probability. The distribution model for the present climatic conditions showed areas with high probability of occurrence across the entire Monte Desert (Fig 8D), suggesting an increment of favourable areas to the southeast in the southern Monte after the LGM. In contrast, for the northern Monte, favourable areas decreased in size and became fragmented compared to the LGM predictions for the same area (Fig 8). The variables that contributed most to all models were Annual Precipitation (BIO12), Mean Temperature of Wettest Quarter (BIO8) and the Mean Annual Temperature (BIO1, see S6 Table).

## Discussion

During Pleistocene glacial cycles, rapid climatic changes caused shifts in the distribution range of many species in South America, leaving an important footprint on their genetic structure [4]. However, the magnitude of such impact at continental scale is difficult to evaluate since many South American regions are still unexplored. The present study constitutes the first botanical phylogeographic study that encompasses the entire Monte phytogeographic province latitudinal extended for about 2,000 km. Statistical phylogeography and niche modelling analyses of the endemic but widespread shrub *Monttea aphylla* reveals how past climate change may have affected the distribution of this focal species, while also furthering our understanding of the evolutionary history of Monte phytogeographic Province.

As expected, historical inferences retrieved by the nuclear and chloroplast molecular markers used in this study, differed due to the different effective sizes between the haploid and diploid markers [68,69]; higher evolution rate and major coalescence times of nuclear than chloroplast marker probably explain the older dating of lineages divergence retrieved by the former while more recent demographic and historical events, as well as a more pronounced phylogeographical structure was better recovered by the chloroplast locus. However, both historical signals were highly concordant and the joint use of these two markers allow us to better disentangle the evolutionary history of the focal species.

*Monttea aphylla* showed a phylogeographic structure that followed a predominantly latitudinal pattern as revealed by the cpDNA genealogical analysis, which recovered two main groups of haplotypes, the northern and southern groups. Accordingly, the multilocus bayesian spatial structure analysis further detected two groups of populations within the northern Monte, and three population groups within the southern Monte, that also followed a latitudinal pattern. Our dating analyses suggest that differentiation of this two main northern and southern lineages of *M*. *aphylla* started around 4 Ma (Pliocene), while diversification within each genetic group occurred mainly in the limit Pliocene-Pleistocene (ca 2.7 Ma). These results are highly congruent with previous estimations performed in south american desert plants (Atacama Desert: [14]; Monte Desert: [15]; Patagonian steppe: [11,12]). Very interestingly these estimates fall after the final uplift of the Andes (c. 5 Ma), which had marked ecological consequences, producing a rain-shadow effect that resulted in the establishment of the extremely xeric regions of South America, such as the Monte and Patagonia [70].

Although the calibration of a molecular clock allows associations between demographic processes and paleoenvironmental events, limitations and biases of this kind of analyses are known due to the use of mutation rates estimated for taxa different from the focus group (reviewed in [71]). However, independent evidence, such as climatic, geographic, demographic and population genetic analyses (i.e. Geneland, SSD, BSP, RRW, PDM), as well as the comparison with other phylogeographic studies in the region, were consistent with our dating results. Future studies should include new independent nuclear markers to increase our adjustment to the different coalescent models used in this study, and to improve our divergence time estimates reducing genealogical uncertainties.

The limit between the northern and the southern genetic group showed a great correspondence with the biogeographic subdivision of the Monte Desert, north and south of 35° S, which coincides with the transition between subtropical and temperate circulation features in South America [25,31]. Differences between northern and southern Monte are mainly explained by changes in topography and rainfall patterns, in particular the annual seasonality (i.e. predominantly summer rainfall in the northern Monte and precipitations equally distributed along the year, in the southern Monte). Interestingly, our climatic reconstructions through time also showed a different historical response to climatic change between both biogeographical areas, being the northern much more stable than the south (see below); thus, probably affecting differentially populations located in northern or southern Monte. In this line, phylogeographic studies in the lizard *Liolaemus darwinii* complex [17] and in the parrot *Cyanoliseus patagonus* [24], both co-distributed species with *M*. *aphylla*, reported the same genetic break, highlighting a possible historical effect shared among these taxa in the Monte desert.

The genetic structure and the inferred historical processes differed for populations located in the northern Monte compared to the southern Monte. Based on cpDNA, in northern localities, the three most frequent haplotypes had allopatric distributions, and population differentiation and nucleotide diversity were higher than the southern group despite the smaller sample size of the former (see Table 1). This pattern is consistent with a phylogeographical study of *Cnemidophorus longicaudata*, a lizard co-distributed with *M*. *aphylla*, which showed greater genetic diversity and more restricted haplotypes in the northern Monte [19]. Furthermore, in the same study certain demographic expansion signal in the northernmost subclade (clade 2–1) was detected as occurs in *M*. *aphylla* for the N-1 group, considering neutrality tests. However, as happens in *M*. *aphylla* with the BSP, for *C*. *longicaudata* not all demographic analyses supported the demographic expansion inference for the northern Monte, revealing a weaker signal of demographic expansion than the southern Monte (see below). Patterns of genetic diversity found in both studies for the northern Monte could be explained by the complex topography of this area, as this ecoregion is dominated by mountain ranges connected by narrow valleys, forming arid habitats like terrestrial islands. Thus, the fragmented nature of this landscape would favour geographic isolation and allopatric differentiation among populations [18]. An alternative but not exclusive explanation for the greater genetic diversity found in the northern compared to southern localities, could be the greater climatic stability of northern Monte during the LIG and LGM as inferred from the PDM.

In contrast, in the southern Monte (south of 35° S), characterized by plains and piedmont [16], higher genetic similarity among populations could be observed. Many haplotypes were shared among populations; in particular H1 of cpDNA, which had a central position in the network, was the most widely distributed haplotype and was present in almost all southern populations. This revealed a great connectivity and recent shared history. Moreover, the pattern of low nucleotide and high haplotype diversity observed in this southern ecoregion indicated the presence of closely related haplotypes, reinforcing the idea of a common recent history of individuals analyzed south of 35° S. Additionally, populations located in the southern boundary of *M*. *aphylla*’s range (south of 38.95° S) were monomorphic for the most common haplotype H1 in cpDNA and nDNA, except for one population that was monomorphic for H4 for cpDNA. Because these same chloroplast haplotypes (H1 and H4) were also found in lowly diverse localities located near the northern limit of southern Monte, low levels of diversity to the south could be the result of founder effects and repeated bottlenecks during a colonization process from the north to the southeast.

Our study takes advantage of a novel development in Bayesian phylogeographic inference, that had been never used in plants before, allowing us to infer the diffusion dynamics of the focal species over continuous space and time (i.e. RRW). On one hand, using this approach, our study gives interesting insights into the relationship that may exists, in terms of timing, between range and demographic expansions and their possible association with past climatic events. In particular, strong evidence of population growth combined with range expansion through time were obtained for populations south of 35° S based on cpDNA. Tempo-spatial reconstruction indicated that such expansion would have started ca. 120,000 years ago, during the LIG, until the beginning of the LGM. After this last cold period, the highest rate of spatial expansion was achieved, but practically without population growth as evidenced in the Bayesian Skyline Plot analysis. These results were consistent with previous studies in vertebrate species co-distributed with *M*. *aphylla* in southern Monte, which showed growth in effective population size during the late Quaternary [17–24]. The beginning of these expansions were estimated at around the beginning of the LIG, and in many cases population growth continued even during the LGM to the Holocene (e.g. [20]). Similarly, phylogeographic studies in the Patagonian steppe (south of Monte Desert, see Fig 1) also reported demographic expansions during the LIG followed by population stasis even during posterior glacial periods [11,12,21–24].

On the other hand, RRW bayesian analysis allowed us to estimate a mean diffusion rate for the two main lineages within *M*. *aphylla*, the northern group would have a slower dispersion rate than the southern group (698,000 km/Ma vs 457,776 km/Ma for the cpDNA and 179,862 km/Ma vs 121,759 km/Ma fo the nDNA); despite confidence intervals of both rates partially overlapped, this different result could be related to the complex topography of the northern Monte, as this ecoregion is dominated by mountain ranges connected by narrow valleys [16]. Future comparative studies including different taxa inhabiting the northern and southern Monte, should allow us to get insights about the influence of the fragmented nature of the northern Monte in the spatio-temporal diffusion process.

In addition, RRW bayesian approach retrieved a mean diffusion rate of *M*. *aphylla* based on chloroplast lineages that varied between 0 to 1.3 meters per year. Although the dispersion of the fruits has not yet been studied, ants have been seen in the field carrying fruit parts to their nests (Baranzelli, personal observations). Interestingly, the mean diffusion rate estimated in this study, is highly coincident with a dispersion rate reported in a previous study including 24 ant-dispersed plant species [72]. On the other hand, diffusion rate estimated for *M*. *aphylla* based on nuclear lineages, varied between 0 to 0.5 per year. Because it is strictly self-incompatible the species depends mainly on oil-collecting bees for efficient pollen flow [73,74]. Future field studies on fruit and pollen dispersion in *M*. *aphylla* are necessary to validate the diffusion rates reported here.

The Bayesian approach provided us a dynamic reconstruction that could be directly compared with similar reconstructions like the PDM approaches. In this case, as a general pattern, our models showed a reduction-expansion from LIG to the present in the environmental suitability of *M*. *aphylla* according with our results of the cpDNA and partially with the nDNA. Despite this, the LGM models (i.e CCSM and MIROC) showed some different predictions on the palaeodistribution of the focal species. On one hand, MIROC’s predictions support cycling retractions of the southern Monte during glacial periods, followed by an expansion toward the Valdez peninsula near the Atlantic coast. On the other hand, CCSM’s predictions also support this retraction but with stables areas in the northeastern of Patagonian Steppe. Distinct predictions obtained under both scenarios are probably due to a differing precipitation levels assumed by both models [65,75]. However, palynological data [27] and palaeo-reconstructions [25,76] suggest that patagonian steppe would have been displaced towards the north during glaciation periods, suggesting a better performance of MIROC model for this region of South America. Accordingly, previous studies in the Monte region showed similar performance in their PDMs, indicating that MIROC models explain better the genetic data rather than the CCSM models [15,20].

Several lines of evidence supported the hypothesis of a decrease in the area of the southern Monte during the last Quaternary glacial cycles, influencing the demographic history of *M*. *aphylla*. These included: a) the high genetic diversity and the presence of exclusive haplotypes between 34° and 38° S (the northern limit of southern Monte) indicating this area as the boundary of the distribution of the species during the cold and dry periods. This restricted zone could be considered a refugium (i.e. place where species has persisted for long periods of time; [1]) during ice ages, and would have been the source area of the southward demographic expansion. Interestingly, this stable area is coincident with localization of putative source areas for spatial expansion in vertebrate species co-distributed with *M*. *aphylla* [20,22]; b) in southern Monte, even under different LGM models (i.e. CCSM and MIROC), the PDM analyses showed a decrease of favourable areas during cold periods, and a subsequent increase of favourable areas during warm periods. Moreover, results from both simulated LGM scenarios indicated that the variable that had the highest contribution to the respective model was the annual winter temperature (S5 table), thus both climatic reconstructions indicated this environmental variable as a limiting condition of *M*. *aphylla* distribution; c) demographic analyses detected demographic growth and a spatial range expansion during the LIG for population located in the southern Monte; a range expansion southeastward begun around this period being the most important colonization after the LGM, when warmer and more humid conditions returned; d) the comparison of independent approaches such as the PDM and the Bayesian reconstruction of the diffusion process (RRW) agreed in the spatial direction of the range expansion.

## Conclusions

In summary, for *M*. *aphylla*, we observed a differential effect of Pleistocene climatic oscillations across the Monte phytogeographic province. In northern populations, greater genetic structure and more relative stable demography of populations appeared to be the result of a more stable climate than in the southern Monte as inferred by the PDM analysis. Although there were signs of demographic growth for N-1, these were weaker than those for the southern Monte. Likewise, range expansion was restricted in space for the northern group, probably associated with the complexity of the topography, and the presence of orographic barriers (e.g. Puna and Prepuna) not found in the southern Monte. On the other hand, Pleistocene glaciations would have generated a shift in the southern distribution edge of *M*. *aphylla* to the north until approximately 38–39° S, where the presence of exclusive haplotypes and high genetically diverse areas confirmed the persistence of the species. During the interglacial periods, new areas were colonized in a southeastern direction toward the Atlantic coast, which was supported by low genetic diversity and haplotype homogeneity in that area.

The great differences observed in paleoclimatic and demographic patterns for the northern and southern Monte may be due to changes in air currents that regulated rainfall between these two Monte ecoregions. To the north, moisture primarily comes from air masses from the Atlantic anticyclone center, while to the south the humidity is determined by the entry of air masses from the Pacific and Atlantic Ocean [25,31]. During cooling glacial periods, the Atlantic coastline shift had an effect on the quantity and quality of the moisture-laden cold masses that came into southern Monte, increasing aridization processes and consequently affecting the population dynamics of Monte biota. Specifically, during the LGM, evidences from pollen and geological studies indicated that rainfall was a 25–35% lower and the dry season longer than at the present time [25]. Accordingly, results of PDM during LGM showed that changes in annual rainfall, annual temperatures and wet months limited the southern distribution of *M*. *aphylla*. Perhaps these changes affected not only the water balance due to the annual rainfall decrease (e.g. 12]), but also other key reproductive processes such as flowering and germination, limiting the southernmost range of the species.

Although this study is the first one considering the whole extension of Monte Desert, our results are congruent with previous phylogeographical studies, including co-distributed animals and a plant species of the Monte Desert and will be useful for elucidating common patterns shared among these taxa. Such common patterns will enable regional and landscape-level patterns of biodiversity to be detected, which are important for understanding macroecology and evolution in a geographical mosaic against a backdrop broadly impacted by geological and paleoclimatic events.

## Supporting information

S1 Doc Document. Permission to publishmap shapefiles.
(DOC)

S1 Fig Location for each set of primers in the *trnQ–rpL16* sequence. (DOC)

S1 Table Sampling sites, geographical coordinates, sample size, elevation, and molecular diversity indexes of the sampled *Monttea aphylla* populations in the South American Arid Diagonal for each data set. (DOC)

S2 Table Outgroup species, locality, coordinates, and voucher number in CORD. (DOC)

S3 Table Internal primer sequences designed for *trnQ–rpL16* region. (DOC)

S4 Table Summary of the results from the species-specific tuning of the ecological niche modelling carried out with 10 replicated runs under the current climatic conditions. (DOC)

S5 Table Curetted database of the presence points for *M*. *aphylla* and the associated absence data used in the potential distribution models. (DOC)

S6 Table Percentage contribution of climatic variables to the past (LIG and LGM) and current potential distributions of *Monttea aphylla*. (DOC)
