# Supplementary figures and images for: Travelling to the south: Phylogeographic spatial diffusion model in Monttea aphylla (Plantaginaceae), an endemic plant of the Monte Desert
Source: PLoS One. 2017 Jun 5;12(6):e0178827. doi: 10.1371/journal.pone.0178827 (PMC5459442; doi:10.1371/journal.pone.0178827)

**S1 Figure**. Location for each set or primers in the *trnQ– rpL16* sequence.


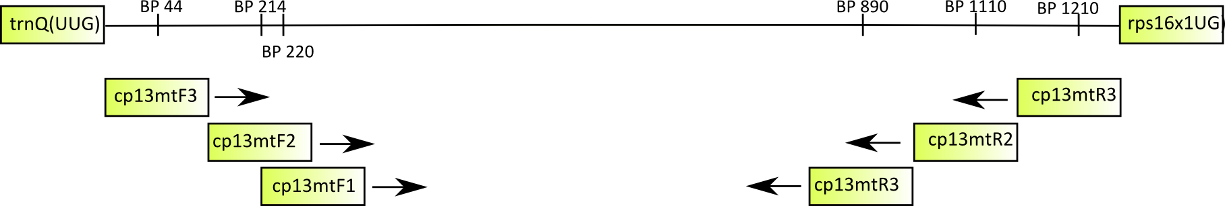

Supplement: S1 Fig — (DOC) [file pone.0178827.s002.doc]
