# Supplementary material for: Travelling to the south: Phylogeographic spatial diffusion model in Monttea aphylla (Plantaginaceae), an endemic plant of the Monte Desert
Source: PLoS One. 2017 Jun 5;12(6):e0178827. doi: 10.1371/journal.pone.0178827 (PMC5459442; doi:10.1371/journal.pone.0178827)
Supplement: S1 Table — (DOC) [file pone.0178827.s003.doc]

**Table S1.** Sampling sites, geographical coordinates, sample size (Nind), elevation (metres), and molecular diversity indexes of the sampled *Monttea aphylla* populations in the South American Arid Diagonal**.**

| **Nloc** | **Sampling site** | **Latitude (°S)** | **Longitude (°S)** | **N_ind_** | **Elevation** | **cpDNA** | | | | | **nDNA** | | | | | **Voucher No in CORD** |
| --- | --- | --- | --- | --- | --- | --- | --- | --- | --- | --- | --- | --- | --- | --- | --- | --- |
|  |  |  |  |  |  | **S** | **K** | **π  (± SD)** | **h (± SD)** | **p (± SD)** | **S** | **K** | **π  (± SD)** | **h (± SD)** | **p (± SD)** |  |
| 1 | Amaicha del Valle | 26,6144 | 65,8419 | 8 | 2353 | 3 | 3 | 0.000326 (0.000304) | 0.4643 (0.2000) | 0.750000 (0.613775) | 1 | 2 | 0.000420 (0.000573) | 0.2857 (0.1964) | 0.285714 (0.340874) | AAC4560 |
| 2 | Barranca Larga | 26,9745 | 66,7416 | 8 | 2531 | 2 | 3 | 0.000217 (0.000234) | 0.4643 (0.2000) | 0.5000 (0.471675) | 0 | 1 | 0.0000 (0.0000) | 0.0000 (0.0000) | 0.0000 (0.0000) | AAC4565 |
| 3 | Punta de Balastro | 26,9802 | 66,1517 | 8 | 2170 | 0 | 1 | 0.0000 (0.0000) | 0.0000 (0.0000) | 0.0000 (0.0000) | 2 | 3 | 0.001206 (0.001092) | 0.6786 (0.1220) | 0.821429 (0.652729) | AAC4562 |
| 4 | El Eje | 27,1178 | 66,8407 | 8 | 2226 | 1 | 2 | 0.000109 (0.000154) | 0.2500 (0.1802) | 0.250000 (0.311294) | 0 | 1 | 0.0000 (0.0000) | 0.0000 (0.0000) | 0.0000 (0.0000) | AAC4514 |
| 5 | Hualfín | 27,2198 | 66,7942 | 8 | 1903 | 1 | 2 | 0.000109 (0.000154) | 0.2500 (0.1802) | 0.250000 (0.311294) | 0 | 1 | 0.0000 (0.0000) | 0.0000 (0.0000) | 0.0000 (0.0000) | AAC4517 |
| 6 | C. de Miranda | 29,3485 | 67,7764 | 8 | 2102 | 1 | 2 | 0.000186 (0.000212) | 0.4286 (0.1687) | 0.428571 (0.428571) | 3 | 4 | 0.6515 (0.1327) | 0.6515 (0.1327) | 1.303030 (0.873753) | AAC4533 |
| 7 | Piedras Pintadas | 29,4259 | 67,8733 | 8 | 1559 | 0 | 1 | 0.0000 (0.0000) | 0.0000 (0.0000) | 0.0000 (0.0000) | 0 | 1 | 0.0000 (0.0000) | 0.0000 (0.0000) | 0.0000 (0.0000) | AAC4498 |
| 8 | Chiflón | 30,207 | 67,564 | 8 | 1179 | 0 | 1 | 0.0000 (0.0000) | 0.0000 (0.0000) | 0.0000 (0.0000) | 3 | 4 | 0.001468 (0.001232) | 0.7778 (0.1100) | 1.000000 (0.739434) | AAC4525 |
| 9 | M. de Hualilán | 30,7356 | 68,9609 | 8 | 1960 | 0 | 1 | 0.0000 (0.0000) | 0.0000 (0.0000) | 0.0000 (0.0000)) | 0 | 1 | 0.0000 (0.0000) | 0.0000 (0.0000) | 0.0000 (0.0000) | AAC4578 |
| 10 | La Ciénaga | 30,9373 | 68,8069 | 8 | 1722 | 0 | 1 | 0.0000 (0.0000) | 0.0000 (0.0000) | 0.0000 (0.0000) | 3 | 3 | 0.000979 (0.000935) | 0.4167 (0.1907) | 0.666667 (0.561202) | AAC4496 |
| 11 | Ea. Maradona | 31,7719 | 68,7907 | 8 | 1332 | 0 | 1 | 0.0000 (0.0000) | 0.0000 (0.0000) | 0.0000 (0.0000) | 3 | 4 | 0.001996 (0.001480) | 0.6026 (0.1306) | 1.358974 (0.896354) | AAC4687 |
| 12 | Cadillo | 31,7843 | 65,1852 | 8 | 709 | 0 | 1 | 0.0000 (0.0000) | 0.0000 (0.0000) | 0.0000 (0.0000) | 1 | 2 | 0.000367 (0.000521) | 0.2500 (0.1802) | 0.250000 (0.311294) | AAC4665 |
| 13 | PN Las Quijadas | 32,5786 | 67,0653 | 8 | 748 | 0 | 1 | 0.0000 (0.0000) | 0.0000 (0.0000) | 0.0000 (0.0000) | 2 | 2 | 0.001044 (0.000964) | 0.3556 (0.1591) | 0.711111 (0.580366) | AAC4679 |
| 14 | Cacheuta | 32,9291 | 69,2274 | 8 | 1567 | 0 | 1 | 0.0000 (0.0000) | 0.0000 (0.0000) | 0.0000 (0.0000) | 0 | 1 | 0.0000 (0.0000) | 0.0000 (0.0000) | 0.0000 (0.0000) | AAC4445 |
| 15 | Agrelo | 33,1075 | 68,8798 | 8 | 973 | 3 | 3 | 0.000326 (0.000304) | 0.4643 (0.2000) | 0.750000 (0.613775) | 1 | 2 | 0.000367 (0.000521) | 0.2500 (0.1802) | 0.250000 (0.311294) | AAC4836 |
| 16 | Goico | 34,6089 | 67,8215 | 8 | 517 | 4 | 3 | 0.000512 (0.000417) | 0.6071 (0.1640) | 0.0000 (0.0000) | 3 | 3 | 0.000979 (0.000935) | 0.4167 (0.1907) | 0.666667 (0.561202) | AAC4767 |
| 17 | Los Tamarindos | 35,2387 | 67,6671 | 8 | 435 | 1 | 2 | 0.000109 (0.000154) | 0.2500 (0.1802) | 0.250000 (0.311294) | 4 | 5 | 0.001993 (0.001561) | 0.8571 (0.1083) | 1.357143 (0.933097) | AAC4766 |
| 18 | Gral. Alvear | 35,2408 | 67,6665 | 8 | 434 | 3 | 2 | 0.000326 (0.000304) | 0.2500 (0.1802) | 0.750000 (0.613775) | 0 | 1 | 0.0000 (0.0000) | 0.0000 (0.0000) | 0.0000 (0.0000) | AAC5020 |
| 19 | Ea. puerta Grande | 36,7557 | 67,3755 | 8 | 436 | 3 | 2 | 0.000698 (0.000525) | 0.5357 (0.1232) | 1.607143 (1.059816) | 1 | 2 | 0.000629 (0.000717) | 0.4286 (0.1687) | 0.000629 (0.000717) | AAC4452 |
| 20 | 25 de Mayo | 37,5019 | 67,7146 | 8 | 420 | 0 | 1 | 0.0000 (0.0000) | 0.0000 (0.0000) | 0.0000 (0.0000) | 1 | 2 | 0.000420 (0.000573) | 0.2857 (0.1964) | 0.285714 (0.340874) | AAC4453 |
| 21 | Puelén | 37,6604 | 67,7371 | 8 | 422 | 3 | 4 | 0.000481 (0.000398) | 0.8214 (0.1007) | 0.000481 (0.000398) | 2 | 3 | 0.001626 (0.001345) | 0.6786 (0.1220) | 1.107143 (0.804239) | AACs/n |
| 22 | Puelches | 38,0811 | 65,6908 | 8 | 288 | 3 | 5 | 0.000683 (0.000516) | 0.8571 (0.1083) | 1.571429 (1.041814) | 2 | 3 | 0.000734 (0.000789) | 0.4643 (0.2000) | 0.500000 (0.471675) | AAC4454 |
| 23 | Curaco | 38,1552 | 67,0504 | 8 | 303 | 3 | 5 | 0.000574 (0.000453) | 0.7857 (0.1508) | 1.321429 (0.914841) | 2 | 3 | 0.000734 (0.000789) | 0.4643 (0.2000) | 0.500000 (0.471675) | AAC4768 |
| 24 | La Adela | 38,9564 | 64,0746 | 8 | 100 | 0 | 1 | 0.0000 (0.0000) | 0.0000 (0.0000) | 0.0000 (0.0000) | 1 | 2 | 0.000326 (0.000480) | 0.2222 (0.1662) | 0.222222 (0.288021) | AAC4579 |
| 25 | Confluencia | 38,9574 | 69,0742 | 8 | 478 | 1 | 2 | 0.000109 (0.000154) | 0.2500 (0.1802) | 0.250000 (0.311294) | 0 | 1 | 0.0000 (0.0000) | 0.0000 (0.0000) | 0.0000 (0.0000) | AAC4462 |
| 26 | Chocón | 39,219 | 68,7715 | 8 | 481 | 2 | 3 | 0.000295 (0.000284) | 0.6071 (0.1640) | 0.678571 (0.574217) | 0 | 1 | 0.0000 (0.0000) | 0.0000 (0.0000) | 0.0000 (0.0000) | AAC4521 |
| 27 | Conesa Norte | 39,4395 | 64,4296 | 8 | 130 | 0 | 1 | 0.0000 (0.0000) | 0.0000 (0.0000) | 0.0000 (0.0000) | 3 | 4 | 0.002210 (0.001578) | 0.6667 (0.0991) | 1.504762 (0.958719) | AAC4580 |
| 28 | Conesa | 40,0237 | 64,4379 | 8 | 86 | 0 | 1 | 0.0000 (0.0000) | 0.0000 (0.0000) | 0.0000 (0.0000) | 0 | 1 | 0.0000 (0.0000) | 0.0000 (0.0000) | 0.0000 (0.0000) | AAC4485 |
| 29 | Ramos Mexia | 40,5192 | 67,3518 | 8 | 506 | 0 | 1 | 0.0000 (0.0000) | 0.0000 (0.0000) | 0.0000 (0.0000) | 0 | 1 | 0.0000 (0.0000) | 0.0000 (0.0000) | 0.0000 (0.0000) | AAC4477 |
| 30 | Valcheta | 40,6392 | 66,2478 | 8 | 213 | 0 | 1 | 0.0000 (0.0000) | 0.0000 (0.0000) | 0.0000 (0.0000) | 0 | 1 | 0.0000 (0.0000) | 0.0000 (0.0000) | 0.0000 (0.0000) | AAC4478 |
| 31 | San Antonio | 40,6697 | 64,9085 | 8 | 26 | 0 | 1 | 0.0000 (0.0000) | 0.0000 (0.0000) | 0.0000 (0.0000) | 1 | 2 | 0.000326 (0.000480) | 0.2222 (0.1662) | 0.222222 (0.288021) | AAC4581 |
| 32 | Sierra Grande | 41,6539 | 65,3338 | 8 | 270 | 0 | 1 | 0.0000 (0.0000) | 0.0000 (0.0000) | 0.0000 (0.0000) | 1 | 2 | 0.000641 (0.000699) | 0.4364 (0.1333) | 0.000641 (0.000699) | AAC4482 |
| 33 | Arroyo verde | 42,0074 | 65,2968 | 8 | 162 | 0 | 1 | 0.0000 (0.0000) | 0.0000 (0.0000) | 0.0000 (0.0000) | 1 | 2 | 0.000753 (0.000761) | 0.5128 (0.0822) | 0.512821 (0.461179) | AAC4582 |
| 34 | Chubut | 42,3704 | 65,1769 | 8 | 105 | 0 | 1 | 0.0000 (0.0000) | 0.0000 (0.0000) | 0.0000 (0.0000) | 0 | 1 | 0.0000 (0.0000) | 0.0000 (0.0000) | 0.0000 (0.0000) | AAC4583 |

S= polymorphic sites; k= number of haplotypes; π= nucleotide diversity; *h*= haplotype diversity; p= mean number of pairwise differences. Voucher numbers deposited at CORD (herbarium of the Museo Botánico de Córdoba) are indicated in the last column. Sampling sites are numbered consecutively, as shown on the map in Fig. 1 and 2
