# Supplementary material for: Travelling to the south: Phylogeographic spatial diffusion model in Monttea aphylla (Plantaginaceae), an endemic plant of the Monte Desert
Source: PLoS One. 2017 Jun 5;12(6):e0178827. doi: 10.1371/journal.pone.0178827 (PMC5459442; doi:10.1371/journal.pone.0178827)
Supplement: S2 Table — (DOC) [file pone.0178827.s004.doc]

**Table S2.** Outgroup species, locality, coordinates, and voucher number in CORD.

| **Outgroup species** | **Collecting site** | **Latitude     (S)** | **Longitude (W)** | **Voucher no.** |
| --- | --- | --- | --- | --- |
| ***Monttea chilensis* Gay** | Paposo, Antofagasta, Chile | 25.0087 | 70.4300 | AAC4903 |
| ***Monttea schickendantzii* Griseb.** | Capilla del Monte, Córdoba, Argentina | 30.8675 | 64.5347 | ACC4518 |
| ***Melosperma andicola Benth.*** | Argentina, Mendoza, Malargüe, Pasando las Leñas, Valle Hermoso | 35.0909 | 70.1295 | 4825 AAC-ANS |
| ***Ourisia coccinea (Miers)*** | Argentina, Neuquén, RP 18 PN Lanín, Lago Ruca Choroi | 39.2376 | 71.1802 | 4790 AAC-ANS |
