# Supplementary material for: Travelling to the south: Phylogeographic spatial diffusion model in Monttea aphylla (Plantaginaceae), an endemic plant of the Monte Desert
Source: PLoS One. 2017 Jun 5;12(6):e0178827. doi: 10.1371/journal.pone.0178827 (PMC5459442; doi:10.1371/journal.pone.0178827)
Supplement: S3 Table — (DOC) [file pone.0178827.s005.doc]

**S3 Table** Internal primer sequences designed for trnQ– rpL16 region, used in this study.

| **Primers**  **name** | **Forward sequences**  **(5´a 3´)** | **Reverse sequences**  **(5´a 3´)** |
| --- | --- | --- |
| cp13mt1 | 5'-CGA TTG AGG CAG GGG TGA TT-3' | 5'- TTG GAA CCG GTA TGG AAT TGA-3' |
| cp13mt2 | 5'-TAT GGG ACG ATT GAG GCA GG-3' | 5'- TGT GAT ATG TGT AGG TGG AAC GA-3' |
| cp13mt3 | 5'-AGT AGG GGA GTC GAT AGG AGT-3' | 5'- TGA ATC TGC ATC TTC AAG TCA GGA-3' |
