# Supplementary material for: Travelling to the south: Phylogeographic spatial diffusion model in Monttea aphylla (Plantaginaceae), an endemic plant of the Monte Desert
Source: PLoS One. 2017 Jun 5;12(6):e0178827. doi: 10.1371/journal.pone.0178827 (PMC5459442; doi:10.1371/journal.pone.0178827)
Supplement: S4 Table — (DOC) [file pone.0178827.s006.doc]

**S4 Table.** Ecological Niche Modeling (ENM).

Based on Pearson correlations, 10 out of the 19 bioclimatic variables were selected: Annual Mean Temperature, Mean Diurnal Range, Isothermality, Temperature Seasonality, Mean Temperature of Wettest Quarter, Mean Temperature of Driest Quarter, Annual Precipitation, Precipitation of Wettest Month, Precipitation of Driest Month and Precipitation of Coldest Quarter

Summary of the results from the species-specific tuning carried on with 10 replicated runs each under the current climatic conditions. The selected regularization parameter was 1.5 marked in bold.

| **Regularization parameter** | **Average test AUC for the replicate runs (SD)** | **AUC differences (training - test AUCs)** | **Average 10 percentile training presence** | **Minimum training presence test omission rates** | **Variable response curves** |
| --- | --- | --- | --- | --- | --- |
| 0,25 | 0,781 (0,076) | 0,115 | 0,273 | 0,071 | irregular |
| 0,50 | 0,796 (0,074) | 0,068 | 0,336 | 0,029 | smooth and regular |
| 0,75 | 0,789 (0,071) | 0,064 | 0,363 | 0,031 | irregular |
| 1,00 | 0,799 (0,082) | 0,042 | 0,385 | 0,029 | irregular |
| 1,25 | 0,804 (0,065) | 0,035 | 0,385 | 0,029 | smooth and regular |
| **1,50** | **0,810 (0,061)** | **0,025** | **0,402** | **0,029** | **smooth and regular** |
| 1,75 | 0,791 (0,075) | 0,039 | 0,421 | 0,031 | smooth and irregular |
| 2,00 | 0,802 (0,090) | 0,030 | 0,418 | 0,031 | regular |
| 2,25 | 0,788 (0,068) | 0,033 | 0,444 | 0,031 | smooth and irregular |
| 2,50 | 0,799 (0,066) | 0,026 | 0,439 | 0,034 | smooth and regular |
| 2,75 | 0,801 (0,071) | 0,028 | 0,418 | 0,032 | regular |
| 3,00 | 0,786 (0,069) | 0,032 | 0,455 | 0,034 | regular |
