# Supplementary material for: Travelling to the south: Phylogeographic spatial diffusion model in Monttea aphylla (Plantaginaceae), an endemic plant of the Monte Desert
Source: PLoS One. 2017 Jun 5;12(6):e0178827. doi: 10.1371/journal.pone.0178827 (PMC5459442; doi:10.1371/journal.pone.0178827)
Supplement: S5 Table — (DOC) [file pone.0178827.s007.doc]

**S5 Table.** Curetted database of the presence points for *M. aphylla* and the associated absence data used in the potential distribution models.

| **SPECIES** | **LATITUDE** | **LONGITUDE** | **CONDITION** |
| --- | --- | --- | --- |
| Monttea aphylla | -32,600890 | -68,922032 | Presence |
| Monttea aphylla | -32,929102 | -69,227441 | Presence |
| Monttea aphylla | -33,107509 | -68,879842 | Presence |
| Monttea aphylla | -35,238738 | -67,667135 | Presence |
| Monttea aphylla | -36,755712 | -67,375472 | Presence |
| Monttea aphylla | -37,501860 | -67,714614 | Presence |
| Monttea aphylla | -37,660414 | -67,737074 | Presence |
| Monttea aphylla | -38,081130 | -65,690826 | Presence |
| Monttea aphylla | -39,068815 | -68,617740 | Presence |
| Monttea aphylla | -38,155240 | -67,050381 | Presence |
| Monttea aphylla | -38,957408 | -69,074198 | Presence |
| Monttea aphylla | -39,041337 | -68,712740 | Presence |
| Monttea aphylla | -40,519241 | -67,351787 | Presence |
| Monttea aphylla | -40,639243 | -66,247834 | Presence |
| Monttea aphylla | -41,653929 | -65,333815 | Presence |
| Monttea aphylla | -40,023690 | -64,437933 | Presence |
| Monttea aphylla | -40,669688 | -64,908485 | Presence |
| Monttea aphylla | -38,956369 | -64,074572 | Presence |
| Monttea aphylla | -31,007284 | -68,759782 | Presence |
| Monttea aphylla | -30,937267 | -68,806854 | Presence |
| Monttea aphylla | -30,719010 | -68,967025 | Presence |
| Monttea aphylla | -29,425854 | -67,873315 | Presence |
| Monttea aphylla | -29,433683 | -67,856143 | Presence |
| Monttea aphylla | -27,117760 | -66,840738 | Presence |
| Monttea aphylla | -26,971310 | -66,738275 | Presence |
| Monttea aphylla | -27,219488 | -66,825914 | Presence |
| Monttea aphylla | -30,206028 | -67,565453 | Presence |
| Monttea aphylla | -29,348520 | -67,776370 | Presence |
| monttea aphylla | -42,007358 | -65,296790 | Presence |
| monttea aphylla | -42,370438 | -65,176909 | Presence |
| Monttea aphylla | -39,218996 | -68,771514 | Presence |
| Monttea aphylla | -26,614356 | -65,841908 | Presence |
| Monttea aphylla | -27,219837 | -66,794175 | Presence |
| Monttea aphylla | -26,980165 | -66,151694 | Presence |
| Monttea aphylla | -30,735607 | -68,960915 | Presence |
| Monttea aphylla | -31,892042 | -65,721189 | Presence |
| Monttea aphylla | -29,300278 | -68,200278 | Presence |
| Monttea aphylla | -32,578610 | -67,065270 | Presence |
| Monttea aphylla | -40,949444 | -66,371667 | Presence |
| Monttea aphylla | -40,914167 | -65,488333 | Presence |
| Monttea aphylla | -40,949444 | -66,371667 | Presence |
| Monttea aphylla | -33,729722 | -66,513056 | Presence |
| Monttea aphylla | -37,261389 | -67,663056 | Presence |
| Monttea aphylla | -33,329444 | -69,235833 | Presence |
| Monttea aphylla | -32,408056 | -69,250278 | Presence |
| Monttea aphylla | -38,246944 | -65,115278 | Presence |
| Monttea aphylla | -40,914167 | -65,488333 | Presence |
| Monttea aphylla | -26,833333 | -66,750000 | Presence |
| Monttea aphylla | -40,914167 | -65,488333 | Presence |
| Monttea aphylla | -40,949444 | -66,371667 | Presence |
| Monttea aphylla | -40,683611 | -64,941389 | Presence |
| Monttea aphylla | -42,308333 | -65,195278 | Presence |
| Monttea aphylla | -41,019722 | -65,435833 | Presence |
| Monttea aphylla | -38,155278 | -66,343333 | Presence |
| Monttea aphylla | -40,914167 | -65,488333 | Presence |
| Monttea aphylla | -38,529444 | -67,466944 | Presence |
| Monttea aphylla | -40,066389 | -64,358056 | Presence |
| Monttea aphylla | -37,381389 | -69,516111 | Presence |
| Monttea aphylla | -41,633333 | -65,383333 | Presence |
| Monttea aphylla | -41,666667 | -65,366667 | Presence |
| Monttea aphylla | -40,066389 | -64,358056 | Presence |
| Monttea aphylla | -33,671389 | -67,319167 | Presence |
| Monttea aphylla | -32,408056 | -69,250278 | Presence |
| Monttea aphylla | -33,358056 | -68,593056 | Presence |
| Monttea aphylla | -32,408056 | -69,250278 | Presence |
| Monttea aphylla | -32,873611 | -69,478611 | Presence |
| Monttea aphylla | -32,873611 | -69,478611 | Presence |
| Monttea aphylla | -33,671389 | -67,319167 | Presence |
| Monttea aphylla | -37,381389 | -69,516111 | Presence |
| Monttea aphylla | -38,529444 | -67,466944 | Presence |
| Monttea aphylla | -32,408056 | -69,250278 | Presence |
| Monttea aphylla | -40,914167 | -65,488333 | Presence |
| Monttea aphylla | -32,408056 | -69,250278 | Presence |
| Monttea aphylla | -37,211111 | -66,568889 | Presence |
| Monttea aphylla | -39,341944 | -64,499167 | Presence |
| Monttea aphylla | -37,381389 | -69,516111 | Presence |
| Monttea aphylla | -40,914167 | -65,488333 | Presence |
| Monttea aphylla | -40,914167 | -65,488333 | Presence |
| Monttea aphylla | -40,772778 | -63,792778 | Presence |
| Monttea aphylla | -40,772778 | -63,792778 | Presence |
| Monttea aphylla | -38,865000 | -68,880000 | Presence |
| Monttea aphylla | -38,070278 | -69,091944 | Presence |
| Monttea aphylla | -38,529444 | -67,466944 | Presence |
| Monttea aphylla | -35,149722 | -67,110556 | Presence |
| Monttea aphylla | -29,400000 | -67,800000 | Presence |
| Monttea aphylla | -31,771859 | -68,790740 | Presence |
| Monttea aphylla | -29,766650 | -67,778367 | Presence |
| Monttea aphylla | -30,207000 | -67,564036 | Presence |
| Monttea aphylla | -32,004461 | -68,771307 | Presence |
| Monttea aphylla | -28,832538 | -67,658894 | Presence |
| Monttea aphylla | -33,649992 | -66,570495 | Presence |
| Monttea aphylla | -39,133338 | -67,679650 | Presence |
| Monttea aphylla | -39,279698 | -65,623838 | Presence |
| Monttea aphylla | -34,022222 | -65,577500 | Absence |
| Monttea aphylla | -36,467778 | -64,179722 | Absence |
| Monttea aphylla | -42,535870 | -63,905950 | Absence |
| Monttea aphylla | -43,056940 | -64,687560 | Absence |
| Monttea aphylla | -42,112500 | -63,808250 | Absence |
| Monttea aphylla | -43,284910 | -65,241200 | Absence |
| Monttea aphylla | -43,317480 | -65,436600 | Absence |
| Monttea aphylla | -41,558860 | -67,260360 | Absence |
| Monttea aphylla | -41,493720 | -68,204800 | Absence |
| Monttea aphylla | -41,298320 | -69,116680 | Absence |
| Monttea aphylla | -40,093340 | -71,298670 | Absence |
| Monttea aphylla | -40,972650 | -71,233540 | Absence |
| Monttea aphylla | -40,744680 | -71,135830 | Absence |
| Monttea aphylla | -38,692960 | -70,712460 | Absence |
| Monttea aphylla | -37,064610 | -70,940430 | Absence |
| Monttea aphylla | -37,064610 | -70,712460 | Absence |
| Monttea aphylla | -36,576100 | -70,191390 | Absence |
| Monttea aphylla | -36,413270 | -69,279510 | Absence |
| Monttea aphylla | -35,631660 | -68,465340 | Absence |
| Monttea aphylla | -36,022460 | -68,693310 | Absence |
| Monttea aphylla | -35,859630 | -69,474920 | Absence |
| Monttea aphylla | -35,045450 | -70,484490 | Absence |
| Monttea aphylla | -34,947750 | -69,246950 | Absence |
| Monttea aphylla | -33,970740 | -69,540050 | Absence |
| Monttea aphylla | -40,777250 | -62,668410 | Absence |
| Monttea aphylla | -39,604840 | -63,287180 | Absence |
| Monttea aphylla | -36,901770 | -64,264190 | Absence |
| Monttea aphylla | -35,859630 | -65,078370 | Absence |
| Monttea aphylla | -35,371120 | -64,003650 | Absence |
| Monttea aphylla | -34,882620 | -64,882960 | Absence |
| Monttea aphylla | -33,807900 | -64,817830 | Absence |
| Monttea aphylla | -33,286830 | -64,752700 | Absence |
| Monttea aphylla | -32,244690 | -64,589860 | Absence |
| Monttea aphylla | -32,733190 | -66,022810 | Absence |
| Monttea aphylla | -32,277250 | -64,785260 | Absence |
| Monttea aphylla | -31,593350 | -64,687560 | Absence |
| Monttea aphylla | -31,821320 | -64,752700 | Absence |
| Monttea aphylla | -31,267680 | -65,045800 | Absence |
| Monttea aphylla | -31,267680 | -65,273770 | Absence |
| Monttea aphylla | -32,407520 | -65,794840 | Absence |
| Monttea aphylla | -39,735100 | -62,668410 | Absence |
| Monttea aphylla | -36,576100 | -64,622430 | Absence |
| Monttea aphylla | -35,827060 | -65,827410 | Absence |
| Monttea aphylla | -35,501390 | -65,208630 | Absence |
| Monttea aphylla | -40,972650 | -63,156910 | Absence |
| Monttea aphylla | -40,549280 | -68,563040 | Absence |
| Monttea aphylla | -28,662310 | -66,218210 | Absence |
| Monttea aphylla | -28,010970 | -66,022810 | Absence |
| Monttea aphylla | -30,551200 | -65,110930 | Absence |
| Monttea aphylla | -27,294500 | -64,427030 | Absence |
| Monttea aphylla | -29,411360 | -64,557290 | Absence |
| Monttea aphylla | -30,355800 | -68,269940 | Absence |
| Monttea aphylla | -30,225530 | -68,628170 | Absence |
| Monttea aphylla | -31,072270 | -69,898290 | Absence |
| Monttea aphylla | -31,300240 | -70,126260 | Absence |
| Monttea aphylla | -29,802160 | -69,800590 | Absence |
| Monttea aphylla | -27,978410 | -66,771850 | Absence |
| Monttea aphylla | -27,750440 | -66,446180 | Absence |
| Monttea aphylla | -29,248520 | -65,013230 | Absence |
| Monttea aphylla | -28,694880 | -65,534300 | Absence |
| Monttea aphylla | -31,723610 | -64,817830 | Absence |
| Monttea aphylla | -25,763850 | -66,218210 | Absence |
| Monttea aphylla | -24,819400 | -65,925110 | Absence |
| Monttea aphylla | -25,405610 | -66,706720 | Absence |
| Monttea aphylla | -25,242780 | -65,110930 | Absence |
| Monttea aphylla | -25,535880 | -67,162660 | Absence |
| Monttea aphylla | -25,405610 | -64,980670 | Absence |
| Monttea aphylla | -25,242780 | -68,237370 | Absence |
| Monttea aphylla | -25,861550 | -68,302500 | Absence |
| Monttea aphylla | -27,164230 | -68,791010 | Absence |
| Monttea aphylla | -31,137410 | -66,511320 | Absence |
| Monttea aphylla | -31,104840 | -66,120510 | Absence |
| Monttea aphylla | -31,397940 | -66,706720 | Absence |
| Monttea aphylla | -30,388370 | -66,120510 | Absence |
| Monttea aphylla | -34,915180 | -69,084110 | Absence |
| Monttea aphylla | -34,752350 | -70,093690 | Absence |
| Monttea aphylla | -26,968830 | -64,622430 | Absence |
| Monttea aphylla | -28,010970 | -64,915530 | Absence |
| Monttea aphylla | -31,397940 | -65,273770 | Absence |
| Monttea aphylla | -31,397940 | -65,664570 | Absence |
| Monttea aphylla | -26,350050 | -65,436600 | Absence |
| Monttea aphylla | -27,131660 | -65,697140 | Absence |
| Monttea aphylla | -25,991820 | -67,390630 | Absence |
| Monttea aphylla | -30,551200 | -68,204800 | Absence |
| Monttea aphylla | -27,001400 | -68,660740 | Absence |
| Monttea aphylla | -25,307910 | -66,120510 | Absence |
| Monttea aphylla | -29,020550 | -69,409780 | Absence |
| Monttea aphylla | -36,738940 | -68,595610 | Absence |
| Monttea aphylla | -35,631660 | -70,028550 | Absence |
| Monttea aphylla | -36,315570 | -65,208630 | Absence |
| Monttea aphylla | -37,422850 | -63,580280 | Absence |
| Monttea aphylla | -38,595260 | -70,321660 | Absence |
| Monttea aphylla | -36,478400 | -70,712460 | Absence |
| Monttea aphylla | -42,438170 | -64,443310 | Absence |
| Monttea aphylla | -41,884530 | -67,830280 | Absence |
| Monttea aphylla | -69,967300 | -40,747130 | Absence |
| Monttea aphylla | -70,193760 | -39,834230 | Absence |
| Monttea aphylla | -68,535020 | -27,842900 | Absence |
| Monttea aphylla | -67,118180 | -26,356950 | Absence |
| Monttea aphylla | -68,880590 | -28,223030 | Absence |
