# Supplementary material for: Travelling to the south: Phylogeographic spatial diffusion model in Monttea aphylla (Plantaginaceae), an endemic plant of the Monte Desert
Source: PLoS One. 2017 Jun 5;12(6):e0178827. doi: 10.1371/journal.pone.0178827 (PMC5459442; doi:10.1371/journal.pone.0178827)
Supplement: S6 Table — (DOC) [file pone.0178827.s008.doc]

**S6 Table** Percentage contribution of climatic variables to the past (LIG and LGM) and current potential distributions of *Monttea aphylla*

| **Scenarios** | **Climatic variable** | **Percentage**  **Contribution (%)** |
| --- | --- | --- |
|  |  |  |
| LIG (120000 years ago) | Annual Mean Temperature | 35.7 |
|  | Mean Temperature of Wettest Quarter | 14.7 |
|  | Annual Precipitation | 12.8 |
|  | Isothermality | 11.7 |
| LGM (21000  years ago; CCSM) | Annual Mean Temperature | 30.2 |
|  | Annual Precipitation | 15.2 |
|  | Mean Temperature of Driest Quarter | 14 |
|  | Mean Temperature of Coldest Quarter | 12.8 |
| LGM (21000  years ago ; MIROC) | Annual Precipitation | 30.6 |
|  | Annual  Temperature | 13.8 |
|  | Mean Temperature of Driest Quarter | 13.4 |
|  | Mean Temperature of Coldest Quarter | 12.5 |
| Current | Annual  Temperature | 41.4 |
|  | Annual Precipitation | 14.8 |
|  | Mean Temperature of Wettest Quarter | 13.7 |
|  | Temperature Seasonality | 11 |
