# Supplementary material for: Travelling to the south: Phylogeographic spatial diffusion model in Monttea aphylla (Plantaginaceae), an endemic plant of the Monte Desert
Source: PLoS One. 2017 Jun 5;12(6):e0178827. doi: 10.1371/journal.pone.0178827 (PMC5459442; doi:10.1371/journal.pone.0178827)
Supplement: S1 Doc — Permission to publishmap shapefiles. (DOC) [file pone.0178827.s001.doc]

S2 Document. Permission to publish map shapefiles

From:	Robert Hijmans <rhijmans@ucdavis.edu>

To:	Matias Baranzelli <matiasbaranzellibc@gmail.com>

Sent:	29 de abril de 2017, 0:40

Subject:Re: Permission to publish maps in PLOS ONE

Dear Dr. Baranzelli,

You have permission to publish the maps you have made in PLoS-One under the Creative Commons Attribution License (CCAL) CC BY 4.0 (http://creativecommons.org/licenses/by/4.0/).

Best regards, Robert Hijmans

--

Robert J. Hijmans

Associate Professor, Department of Environmental Science and Policy

Chair, Geography Graduate Group

Director, Geospatial and Farming Systems Consortium, Feed the Future Sustainable Intensification Innovation Lab

University of California, Davis

2001 Wickson Hall

(530) 752-6555

On Wed, Apr 26, 2017 at 10:03 AM, Matias Baranzelli <matiasbaranzellibc@gmail.com> wrote:

Dear Dr. Hijmans,

I recently submitted a manuscript for publication to PLOS ONE using shapefiles available on two of your websites, Global Administrative Areas (http://gadm.org/country) and DIVA-GIS (http://www.diva-gis.org/Data). As a part of our revise and resubmit, PLOS ONE has requested we seek permission to publish these materials under the specific Creative Commons Attribution License (CCAL) CC BY 4.0.

I request permission for the open-access journal PLOS ONE to publish maps under the Creative Commons Attribution License (CCAL) CC BY 4.0 (http://creativecommons.org/licenses/by/4.0/). Please be aware that this license allows unrestricted use and distribution, even commercially, by third parties. Please reply and provide explicit written permission to publish these maps under a CC BY license.

Best,

--

Dr. Matias C. Baranzelli

Laboratorio de Ecología Evolutiva y Biología Floral. IMBIV - CONICET

Av. Vélez Sarfield 1611. Edificio de Investigaciones Biológicas y Tecnológicas.

Universidad Nacional de Córdoba
